# *Lomatium* Species of the Intermountain Western United States: A Chemotaxonomic Investigation Based on Essential Oil Compositions

**DOI:** 10.3390/plants14020186

**Published:** 2025-01-11

**Authors:** William N. Setzer, Ambika Poudel, Prabodh Satyal, Kathy Swor, Clinton C. Shock

**Affiliations:** 1 Department of Chemistry, University of Alabama in Huntsville, Huntsville, AL 35899, USA; 2 Aromatic Plant Research Center, 230 N 1200 E, Suite 100, Lehi, UT 84043, USApsatyal@aromaticplant.org (P.S.); 3 Independent Researcher, 1432 W. Heartland Dr., Kuna, ID 83634, USA; 4 Department of Crop and Soil Science, Oregon State University, Ontario, OR 97914, USA; clinton.shock@gmail.com

**Keywords:** anomalum, dissectum, grayi, nudicaule, packardiae, papilioniferum, triternatum, chemotaxonomy, enantiomers

## Abstract

*Lomatium* is a genus of 98 species, widely distributed in western North America. This work presents a chemometric analysis of the essential oils of seven species of *Lomatium* (*L. anomalum*, *L. dissectum* var. *dissectum*, *L. multifidum*, *L. nudicaule*, *L. packardiae*, *L. papilioniferum*, and *L. triternatum* var. *triternatum*) from the intermountain western United States (Oregon and Idaho). The essential oils were obtained by hydrodistillation and analyzed by gas chromatographic methods. *Lomatium packardiae* essential oil can be characterized as limonene-rich, *L. anomalum* is a species rich in sabinene and α-pinene, and *L. multifidum* essential oils were rich in myrcene, while *L. dissectum* var. *dissectum* essential oils were dominated by octyl acetate and decyl acetate, *L. papilioniferum* essential oils from western Idaho had high *p*-cymene and 2-methyl-5-(1,2,2-trimethylcyclopentyl)phenol concentrations, while those from Oregon had relatively high β-phellandrene and sedanenolide levels. The essential oils of *L. triternatum* var. *triternatum* were too variable to confidently assign a chemical type. The major components in the *L. nudicaule* essential oils were β-phellandrene (16.0–45.7%), (*Z*)-ligustilide (5.6–47.1%), (*E*)-β-ocimene (3.3–9.9%), and δ-3-carene (0.2–12.6%). The enantiomeric distributions of α-pinene, camphene, sabinene, β-pinene, limonene, and linalool were also utilized to discriminate between the *Lomatium* taxa. There are not enough consistent data to properly characterize *L. triternatum* var. *triternatum* or the Oregon *L. papilioniferum* essential oils. Additional research is needed to confidently describe the chemotype(s) of these species.

## 1. Introduction

The genus *Lomatium* Raf. (Apiaceae) comprises around 98 species, which are distributed in western North America [1]. The genus is part of one of the largest plant radiations in North America, the Perennial Endemic North American Apiaceae (PENA) clade [2,3]. Several species of *Lomatium* have been used by Native Americans of the Pacific Northwest as medicines as well as food [4]. As part of our continuing interest in essential oils from aromatic and medicinal plants in the intermountain western United States, the purpose of this work is to examine the essential oil compositions, including the enantiomeric distributions of chiral terpenoids, of *Lomatium* species growing in eastern Oregon and western Idaho.

The *Lomatium triternatum* (nineleaf biscuitroot) complex has a widespread distribution from British Columbia, south into northern California, and east to Montana, Wyoming, Colorado, and New Mexico [5,6]. It is a perennial herb (ca. 20–80 cm tall) growing from a taproot. The leaves are basal with petioles 8–20 cm long, leaves divided 1–3 times. The inflorescence is a loose flat umbel of yellow flowers on stalks 3–10 cm long. The seeds are flat with five ribs and thin wings on the sides [5,7,8]. The taxonomy of the *L. triternatum* is complex, is not well delineated, and is in flux [2,3,9,10]. These include, but are not necessarily limited to, *Lomatium triternatum* (Pursh) J.M. Coult. & Rose (which includes the infraspecific taxa *L. triternatum* var. *triternatum*, *Lomatium triternatum* f. *lancifolium* (H. St. John) H. St. John, *Lomatium triternatum* subsp. *platycarpum* (Torr.) Cronquist, *Lomatium triternatum* var. *brevifolium* (J.M. Coult. & Rose) Mathias, and *Lomatium triternatum* var. *macrocarpum* (J.M. Coult. & Rose) Mathias), *Lomatium anomalum* Jones ex J.M. Coult. & Rose, and *Lomatium packardiae* Cronquist [1]. As far as we are aware, there are no reports on the essential oils of *L. triternatum*. The purpose of this research is to examine the hypothesis that the volatile phytochemistry of the different taxa of *L. triternatum* will delineate the members of the complex.

*Lomatium grayi* (J.M. Coult. & Rose) J.M. Coult. & Rose (Gray’s biscuitroot) is a large (up to 60 cm tall) perennial herb with a branched basal stem structure and finely divided leaves with a pungent odor. The inflorescence is an umbel with numerous yellow flowers [11]. The native range of *L. grayi* is east of the Cascades in southern British Columbia, Washington and Oregon, northern Nevada, western Idaho, Utah, western Wyoming, western Colorado, and northwestern New Mexico [12]. However, the *Lomatium grayi* complex is morphologically diverse across its range. Alexander and co-workers have proposed splitting *L. grayi* into four species based on morphometric analysis [13]. These include *Lomatium papilioniferum* J.A. Alexander & Whaley (distributed east of the Cascades in southern British Columbia, Washington, Oregon, northern Nevada, and western Idaho), *Lomatium klickitatense* J.A. Alexander & Whaley (found in Klickitat County, Washington, and surrounding areas), *Lomatium depauperatum* (M.E. Jones) J.A. Alexander & Whaley (syn. *Lomatium grayi* var. *depauperatum* (M.E. Jones) Mathias) (ranges in western Utah and eastern Nevada), and *Lomatium grayi* (in eastern Idaho, eastern Utah, southwestern Wyoming, and western Colorado). In Idaho, *L. papilioniferum* is found in western and central Idaho while *L. grayi* is found only in southeastern Idaho.

*Lomatium dissectum* (fernleaf biscuitroot) is a perennial herb. The inflorescence is an umbel of numerous small maroon red flowers; the leaves are ternate-pinnately dissected, 15–35 cm wide with a 3–30 cm petiole. The fruit is oblong-ovate to elliptic, 12–16 mm long, with thick lateral wings. The plant occurs in western North America, northern California, north into Washington, and east into Idaho [14,15]. *Lomatium dissectum* (Nutt.) Mathias & Constance var. *dissectum*, *Lomatium dissectum* var. *multifidum* (Nutt.) Mathias & Constance, and *Lomatium dissectum* var. *eatonii* (J.M. Coult. & Rose) Cronquist had been treated as varieties of *L. dissectum*. However, they are currently treated as separate species, *Lomatium dissectum* (Nutt.) Mathias & Constance and *Lomatium multifidum* (Nutt.) R.P. McNeill & Darrach (syn. *Lomatium dissectum* var. *multifidum* (Nutt.) Mathias & Constance and *Lomatium dissectum* var. *eatonii* (J.M. Coult. & Rose) Cronquist) [14].

*Lomatium multifidum* is a perennial herb, growing up to 1.2 m tall. The leaves are triangular-ovate to round and ternate-pinnately dissected. The inflorescence is an umbel of numerous small yellow flowers; the fruit is dorsally compressed, with the lateral wings usually well developed. The plant occurs in arid regions of the western United States (Arizona, California, Colorado, Idaho, Montana, Nevada, Oregon, Utah, Washington, and Wyoming) and into southwestern Canada (British Columbia, Alberta, and Saskatchewan) [16,17].

*Lomatium nudicaule* (Nutt.) J.M. Coult. & Rose (barestem biscuitroot) is a perennial forb with a stout taproot. The plant can reach a height of 20–45 cm; the leaves are compound ternate to biternate, leaflets are oval, 2–5 cm long; the inflorescence is an umbel with yellow flowers; fruits are 8–12 mm long, 2–5 mm wide, with 0.5 mm wide wings. The plant ranges from southern British Columbia, south through Washington and Oregon and into northern California, and east into Idaho Nevada and northwestern Utah [5,18]. There have been no previous reports on the essential oil of *L. nudicaule*.

**Figure 1:**
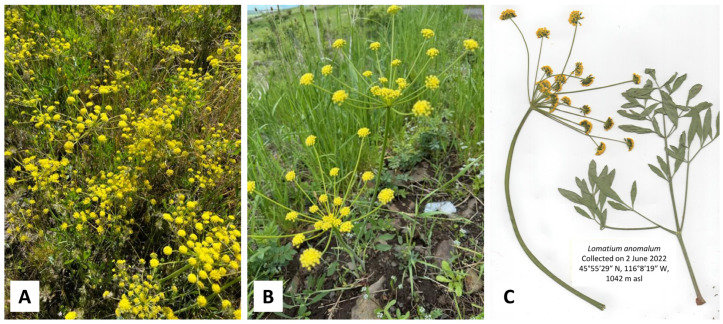
*Lomatium anomalum* Jones ex J.M. Coult. & Rose. (**A**) Several plants at time of collection (2 June 2022, photograph by K. Swor). (**B**) Photograph of plants at time of collection (30 May 2024, photograph by K. Swor). (**C**) Scan of pressed plant.

**Figure 2:**
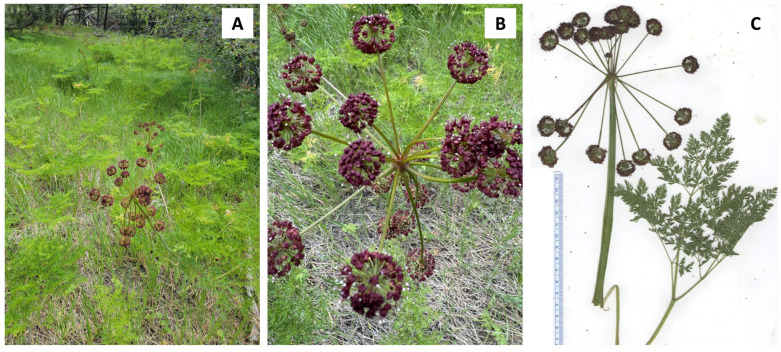
*Lomatium dissectum* (Nutt.) Mathias & Constance. (**A**) Several plants (photograph by W.N. Setzer). (**B**) Closeup of the inflorescence (photograph by K. Swor). (**C**) Scan of the pressed plant.

**Figure 3:**
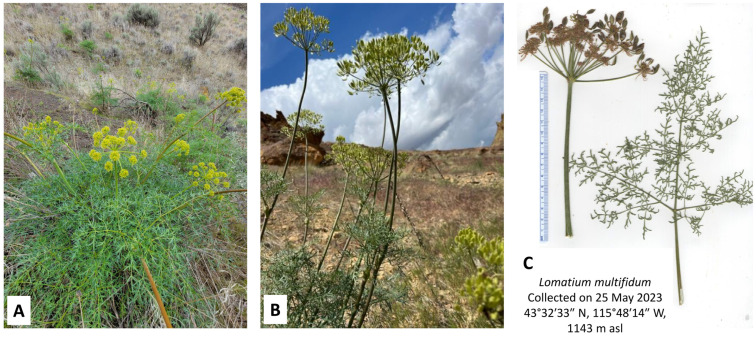
*Lomatium multifidum* (Nutt.) R.P. McNeill & Darrach. (**A**) Flowering stage (photograph by K. Swor). (**B**) Fruiting stage (photograph by W.N. Setzer). (**C**) Scan of the pressed plant.

**Figure 4:**
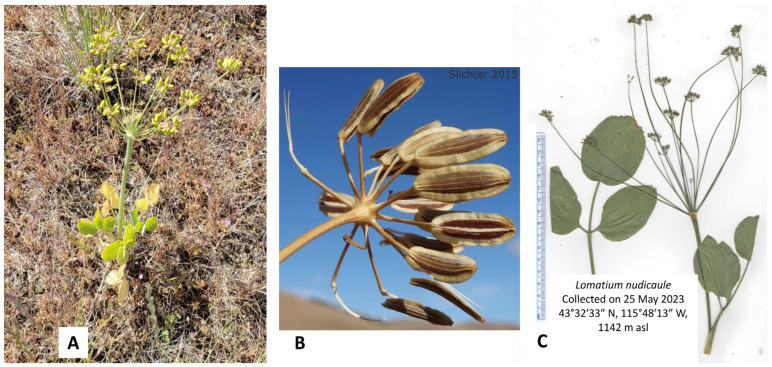
*Lomatium nudicaule* (Nutt.) J.M. Coult. & Rose. (**A**) Photograph of the plant at the time of collection (21 May 2024, photograph by W.N. Setzer). (**B**) Closeup of the fruits (© Paul Schlichter, with permission [19]). (**C**) Scan of the pressed plant.

**Figure 5:**
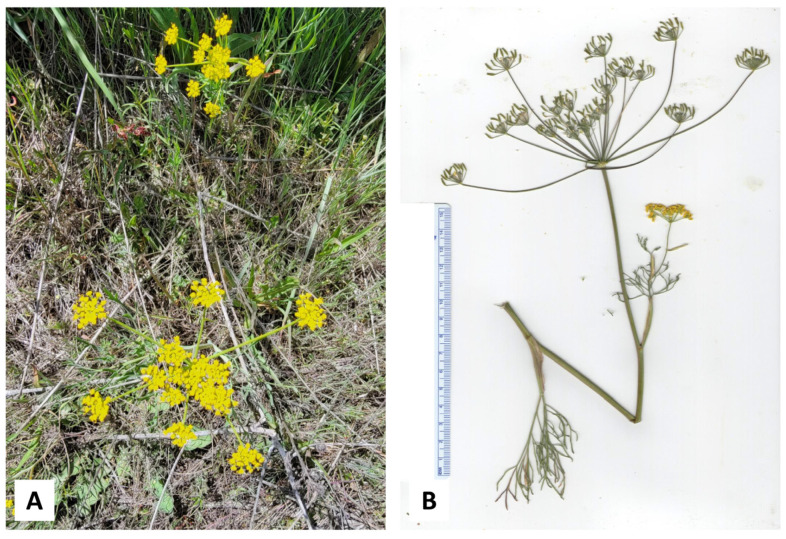
*Lomatium packardiae* Cronquist. (**A**) Photograph of the plant (W.N. Setzer). (**B**) Scan of the pressed plant material.

**Figure 6:**
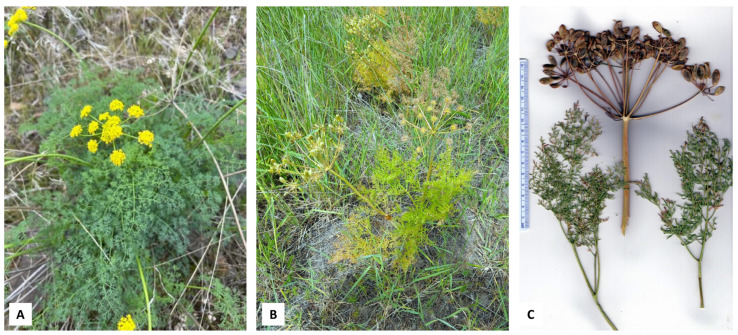
*Lomatium papilioniferum* J.A. Alexander & Whaley. (**A**) Flowering stage (photo by K. Swor). (**B**) Fruiting stage (photo by W.N. Setzer). (**C**) Scan of pressed plant material.

**Figure 7:**
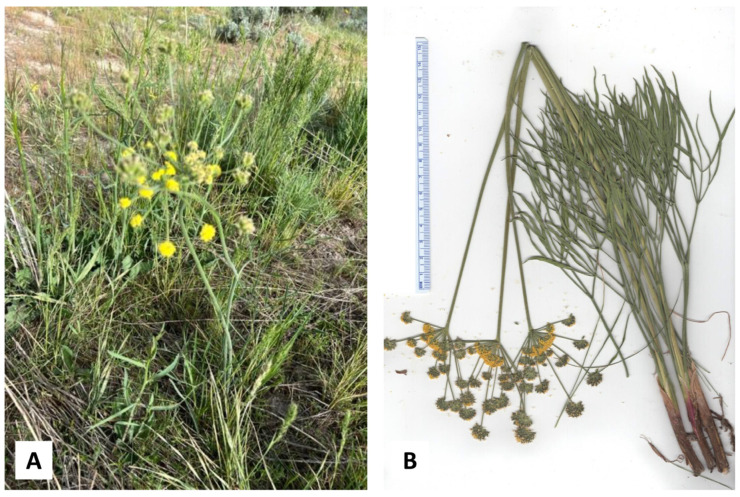
*Lomatium triternatum* (Pursh) J.M. Coult. & Rose var. *triternatum*. (**A**) Photograph of the plant (K. Swor). (**B**) Scan of the pressed plant material.

In this work, we present the essential oil compositions of *L. anomalum* (Figure 1), *L. dissectum* (Figure 2), *L. multifidum* (Figure 3), *L. nudicaule* (Figure 4), *L. packardiae* (Figure 5), *L. papilioniferum* (Figure 6), and *L. triternatum* var. *triternatum* (Figure 7). The purpose of this study is to characterize the volatile components of understudied *Lomatium* species, including enantiomeric distributions of chiral terpenoid components.

## 2. Results and Discussion

There have been several investigations on *Lomatium* essential oils reported in the literature. A summary of the major components is listed in Table 1.

### 2.1. Lomatium anomalum (L. triternatum Complex)

Three samples of *L. anomalum* were collected near Grangeville, western Idaho. Hydrodistillation gave colorless or pale yellow essential oils in yields of 1.57–1.68%. The gas chromatographic results are summarized in Table 2. The essential oils were dominated by sabinene (48.0–49.9%) and α-pinene (21.9–37.6%).

### 2.2. Lomatium packardiae (L. triternatum Complex)

Four samples of *L. packardiae* were collected, two from the Arrowrock Reservoir area (Idaho) and two from the Midvale area (Idaho). The essential oil yields ranged from 1.04% to 1.92%. The essential oil compositions are summarized in Table 3. The major components in the essential oils of *L. packardiae* were limonene (48.6–72.2%), (Z)-ligustilide (12.3–19.1%), and β-phellandrene (4.4–6.2%).

### 2.3. Lomatium triternatum var. triternatum (L. triternatum Complex)

Three individual samples of *L. triternatum triternatum* were collected near Prairie, Idaho. The chemical compositions of the *L. triternatum triternatum* essential oils are summarized in Table 4. Although the three samples were collected from the same location on the same day, there was remarkable variation in the essential oil compositions. For example, monoterpene hydrocarbons ranged from a high of 62.2% in sample Ltt#1 to a low of 13.8% in sample Ltt#2, while oxygenated monoterpenoids were highest in Ltt#2 (39.1%) but lowest in Ltt#3 (2.8%). These are reflected in β-phellandrene concentrations (48.5% and 29.4% in Ltt#1 and Ltt#3, respectively, but only 1.7% in Ltt#2) and myrcene concentrations (12.7% and 14.1% in Ltt#1 and Ltt#3, respectively, but 2.9% in Ltt#2). On the other hand, the cryptone concentration was highest in Ltt#2 (17.9%) compared to either Ltt#1 or Ltt#3 (3.7% and 0.8%). It is not clear what effects may have resulted in these vast differences.

**Figure 8:**
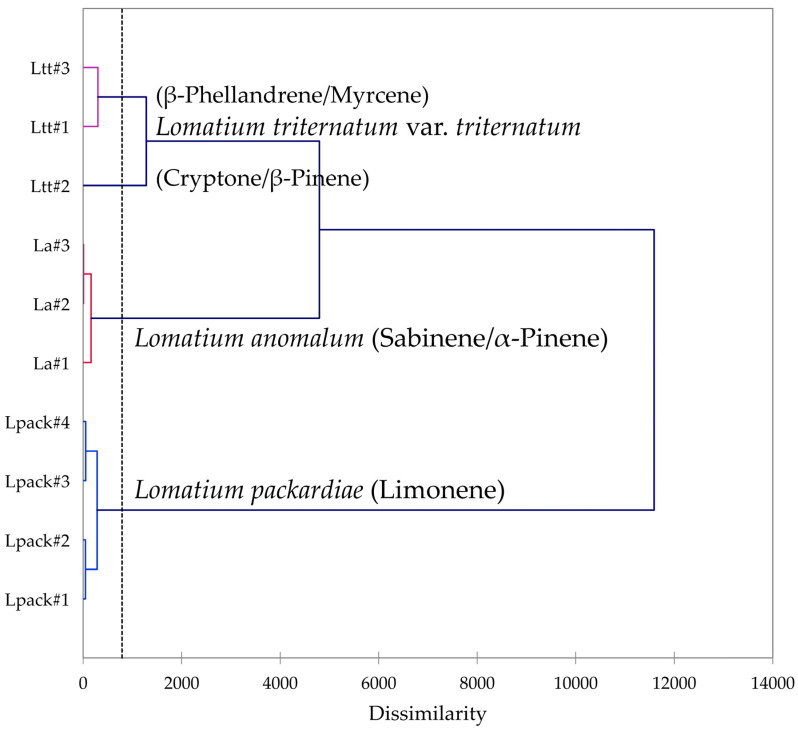
Dendrogram obtained by hierarchical cluster analysis (HCA) of essential oil compositions (major essential oil components) of members of the *Lomatium triternatum* complex.

**Figure 9:**
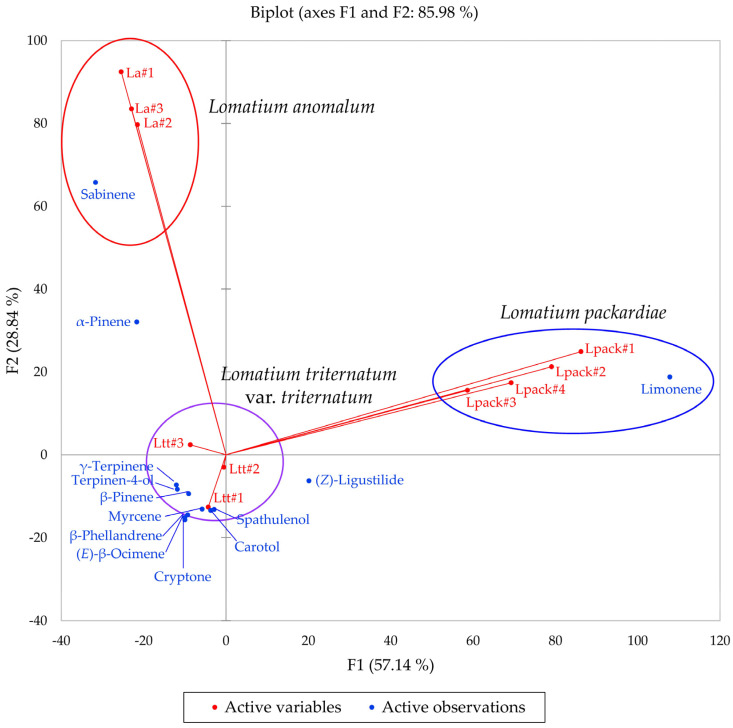
The bidimensional plot of the first two components (F1 and F2) from principal component analysis (PCA) of members of the *Lomatium triternatum* complex, based on major components in their essential oils. La = *Lomatium anomalum*, Lpack = *Lomatium packardiae*, Ltt = *Lomatium triternatum* var. *triternatum*.

Multivariate analyses were performed using the essential oil compositions of *L. anomalum*, *L. packardiae*, and *L. triternatum*, three members of the *L. triternatum* complex, in order to visualize the chemical relationships between the three taxa. A hierarchical cluster analysis (HCA, Figure 8) confirms the large degree of dissimilarity between *L. anomalum*, *L. packardiae*, and *L. triternatum*. The HCA clearly separates the three taxa, the limonene-rich *L. packardiae*, the sabinene/α-pinene *L. anomalum*, and the *L. triternatum* group. The *L. triternatum* group is further subdivided in a β-phellandrene/myrcene type and a cryptone/β-pinene type. A principal component analysis (PCA, Figure 9) corroborates the groupings and their chemical correlations.

### 2.4. Lomatium dissectum (Lomatium dissectum Complex)

Five different individual plants were collected near Grangeville, Idaho. Hydrodistillation of the samples gave colorless essential oils in yields ranging from 1.94% to 2.74%. The chemical compositions of the essential oils are compiled in Table 5. Interestingly, terpenoids were found in very small quantities in *L. dissectum* essential oils. Fatty-acid-derived compounds, however, were the major components, including octyl acetate (37.8–48.4%), decyl acetate (33.9–45.8%), and decanol (9.8–18.4%). These results show some qualitative similarities to that reported by Bairamian and co-workers [22] on a sample from northern California. However, quantitatively, the samples are very different. The California sample had 5.3% octyl acetate, 3.2% decyl acetate, and 1.2% decanol, but a large concentration of palmitic acid (15.3%), which was found in only trace quantities in the samples from Idaho.

### 2.5. Lomatium multifidum (Lomatium dissectum Complex)

A total of 12 samples of *L. multifidum* were collected from locations in eastern Oregon and western Idaho. The essential oils obtained were colorless to yellow with yields ranging from 1.60% to 6.15%. The chemical compositions of the *L. multifidum* essential oils are shown in Table 6. A total of 206 compounds were identified in the essential oils of *L. multifidum*, which accounted for 87.9% to 99.3% of the total compositions. There was some variation in the compositions of the essential oils. The major components were myrcene (12.5–54.1%), (*E*)-β-ocimene (0.3–37.4%), limonene (0.7–14.0%), α-bisabolol (0.0–26.3%), and β-phellandrene (trace-21.3%). In contrast, a sample of *L. multifidum* (reported as *Lomatium dissectum* var. *multifidum*) from southern California showed 6.0% myrcene, 1.0% (*E*)-β-ocimene, 3.3% limonene + β-phellandrene, and 0.1% α-bisabolol [22].

**Figure 10:**
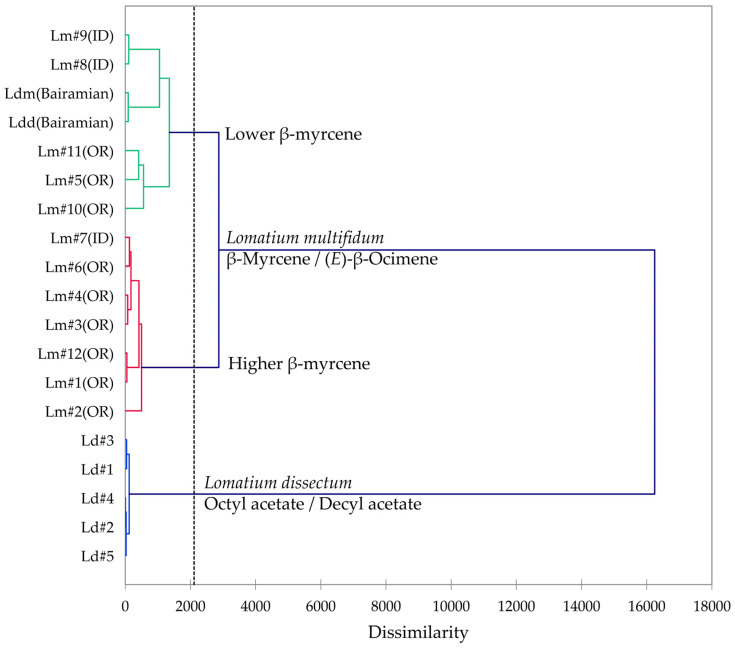
Dendrogram obtained by hierarchical cluster analysis (HCA) of essential oil compositions (major essential oil components) of members of the *Lomatium dissectum* complex. Lm (OR) = *Lomatium multifidum* from eastern Oregon, Lm (ID) = *Lomatium multifidum* from western Idaho, Ld = *Lomatium dissectum* (from western Idaho), Ldd (Bairamian) = *Lomatium dissectum* var. *dissectum* from reference [22], Ldm (Baiaramian) = *Lomatium dissectum* var. *multifidum* from reference [22].

**Figure 11:**
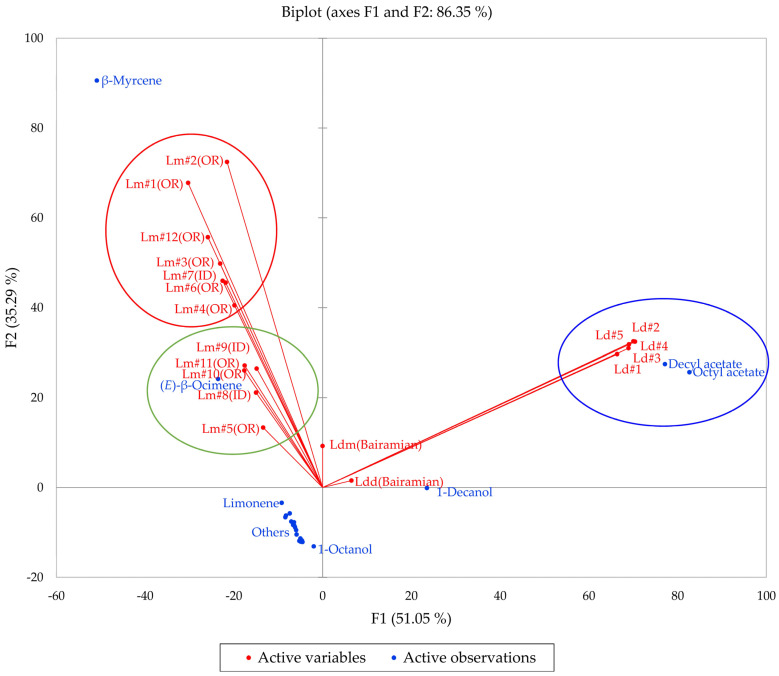
The bidimensional plot of the first two components (F1 and F2) from principal component analysis (PCA) of members of the *Lomatium dissectum* complex, based on major components in their essential oils. Lm (OR) = *Lomatium multifidum* from eastern Oregon, Lm (ID) = *Lomatium multifidum* from western Idaho, Ld = *Lomatium dissectum* (from western Idaho), Ldd (Bairamian) = *Lomatium dissectum* var. *dissectum* from reference [22], Ldm (Baiaramian) = *Lomatium dissectum* var. *multifidum* from reference [22].

Multivariate analyses (HCA and PCA) were carried out in order to visualize the chemical differences and associations in the essential oils of the two members of the *L. dissectum* complex (*L. dissectum* and *L. multifidum*). The HCA dendrogram and the PCA biplot are shown in Figure 10 and Figure 11, respectively. The HCA shows two major clusters: (1) a cluster made up of *L. dissectum* samples, dominated by octyl acetate and decyl acetate, and (2) a cluster with β-myrcene and (*E*)-β-ocimene as defining components and populated by *L. multifidum* samples. The *L. dissectum* and *L. multifidum* samples from Bairamian and co-workers [22] were included in the HCA for comparison. The *L. multifidum* cluster can be subdivided further depending on the concentrations of β-myrcene. The PCA biplot also shows three groupings: (1) the *L. dissectum* group, (2) the *L. multifidum* high β-myrcene group, and (3) the *L. multifidum* less β-myrcene group. The two samples from Bairamian and co-workers (*L. dissectum* var. *multifidum* and *L. dissectum* var. *dissectum*) are separated from the other groups.

### 2.6. Lomatium nudicaule

Seven samples of *L. nudicaule* were collected from three sites in western Idaho. The colorless to pale yellow essential oils were obtained in yields of 0.15% to 3.01%. The essential oil compositions are presented in Table 7. A total of 109 compounds were identified in the essential oils, accounting for 90.4% to 98.7% of the total compositions. The major components in the *L. nudicaule* essential oils were β-phellandrene (16.0–45.7%), (*Z*)-ligustilide (5.6–47.1%), (*E*)-β-ocimene (3.3–9.9%), δ-3-carene (0.2–12.6%), myrcene (0.7–6.1%), cryptone (0.3–7.7%), and germacrene B (0.2–9.3%).

### 2.7. Lomatium papilioniferum (Lomatium grayi Complex)

A total of eight samples of *L. papilioniferum* were collected from north-central Oregon, along the Columbia River (four samples), and from western Idaho (four samples). The plants gave colorless to yellow essential oils (0.20–3.33% yield). The essential oil compositions showed notable differences between the Oregon samples and the Idaho samples (Table 8). Essential oils from both collection locations were generally rich in *p*-cymene (3.1–47.8% and 20.4–22.9%) and γ-terpinene (0.1–30.9% and 7.3–15.1%) for the Oregon and Idaho samples, respectively. However, sedanenolide (1.5–10.8%), myrcene (3.1–27.5%), and (*E*)-β-ocimene (0.7–7.2%) were relatively abundant in the Oregon samples but were either not observed (sedanenolide) or found in only small quantities (myrcene and (*E*)-β-ocimene) in the Idaho samples. Conversely, 2-methyl-5-(1,2,2-trimethylcyclopentyl)phenol (24.9–31.5%) and cuparene (3.5–6.0%) were abundant in the Idaho samples but not observed in the Oregon samples.

**Figure 12:**
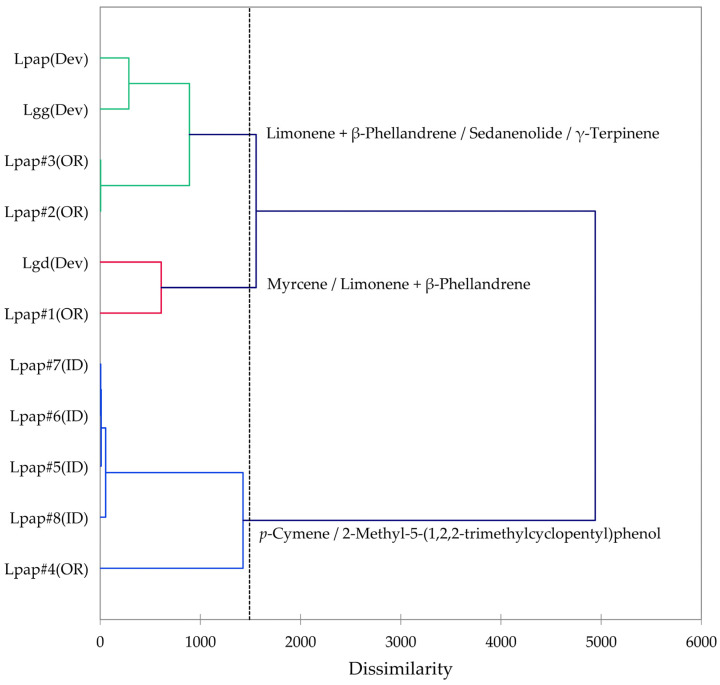
Dendrogram obtained by hierarchical cluster analysis (HCA) of essential oil compositions (major essential oil components) of members of the *Lomatium grayi* complex. Lpap (OR) = *Lomatium papilioniferum* from northern Oregon, Lpap (ID) = *Lomatium papilioniferum* from western Idaho, Lpap(Dev) = *Lomatium* “new species” (*L. papilioniferum*) from reference [24], Lgg(Dev) = *Lomatium grayi* var. *grayi* from reference [24], Lgd(Dev) = *Lomatium grayi* var. *depauparatum* from reference [24].

**Figure 13:**
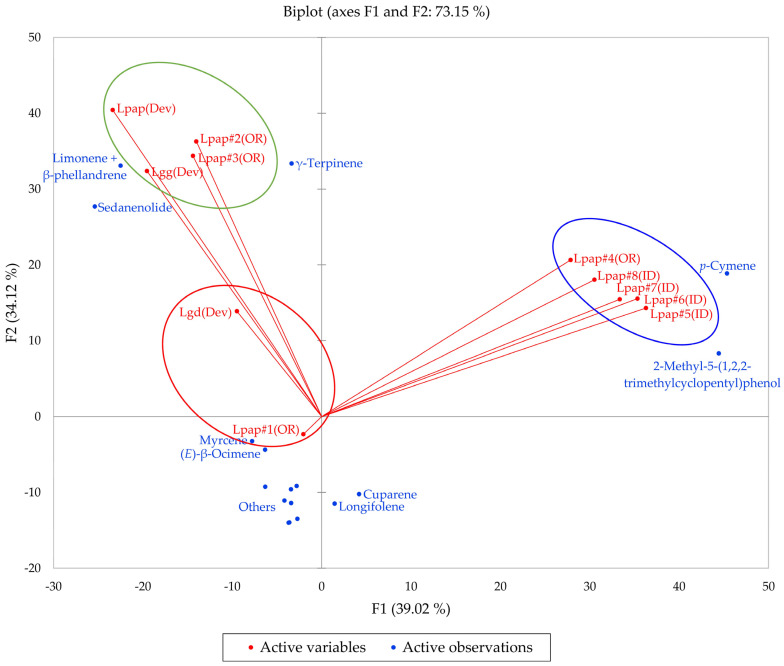
The bidimensional plot of the first two components (F1 and F2) from principal component analysis (PCA) of members of the *Lomatium grayi* complex, based on major components in their essential oils. Lpap (OR) = *Lomatium papilioniferum* from northern Oregon, Lpap (ID) = *Lomatium papilioniferum* from western Idaho, Lpap(Dev) = *Lomatium* “new species” (*L. papilioniferum*) from reference [24], Lgg(Dev) = *Lomatium grayi* var. *grayi* from reference [24], Lgd(Dev) = *Lomatium grayi* var. *depauparatum* from reference [24].

Based on the morphological characteristics as well as the geographical ranges suggested by Alexander et al. [13], the *L. grayi* samples in this work were identified as *L. papilioniferum*. Dev and co-workers [24] analyzed three taxa of the *L. grayi* complex, *L. grayi* var. *grayi*, *L. grayi* var. *depauparatum*, and *L. grayi* “new variety”, which is presumably *L. papilioniferum* based on the location of the collection site (northern Nevada). In order to compare the chemical compositions of the *L. grayi* complex (in this work and [24]), both HCA and PCA were carried out (Figure 12 and Figure 13).

The HCA shows two major groupings (samples #1, #2, and #3 from Oregon and the three *L. grayi* samples from Dev et al. [24]). This group can be further divided into two groups, a limonene + β-phellandrene/sedanenolide/γ-terpinene group and a myrcene/limonene + β-phellandrene group. The second major group, with a very different chemical profile, is dominated by *p*-cymene and 2-methyl-5-(1,2,2-trimethylcyclopentyl)phenol. It is not clear what factors are responsible for the chemical differences observed between the Oregon *L. papilioniferum* samples; these were collected on the same day (17 April 2023) from the same location (along the Columbia River in north-central Oregon). The four *L. papilioniferum* samples from Idaho, collected on the same day (21 May 2024) from the same location (western Idaho), showed very similar chemical profiles. The PCA verifies the HCA. There is a group that correlates strongly with limonene + β-phellandrene, γ-terpinene, and sedanenolide (Lpap#2, and #3, Lpap(Dev), and Lgg(Dev)), a group that correlates strongly with *p*-cymene, and two individual samples (Lgd(Dev and Lpap#1)). The *p*-cymene group may constitute a discrete chemotype of *L. papilioniferum*, while the volatile phytochemical profiles displayed by the Oregon samples are complicated and unresolved.

### 2.8. Analysis of Variance

Analysis of variance (ANOVA) examinations were carried out to identify statistically significant differences in percentages of essential oil components (Table 9). Analyses of the essential oil compositions of *L. anomalum*, *L. packardiae*, and *L. triternatum* var. *triternatum* allow for discrimination between the members of the *L. triternatum* complex. *Lomatium packardiae* essential oils contain a significantly higher concentration of limonene (60.9% ± 10.1%) than the other essential oils, including *L. anomalum* (1.2% ± 0.5%) or *L. triternatum triternatum* (2.5% ± 2.0%). (*Z*)-Ligustilide concentrations were significantly higher in *L. packardiae* (16.2% ± 3.0%) than either *L. anomalum* (0.4% ± 0.4%) or *L. triternatum triternatum* (not observed). On the other hand, *L. anomalum* essential oils had significantly higher concentrations of both sabinene (48.7% ± 1.0%) and α-pinene (27.7% ± 8.6%) than the other *Lomatium* essential oils. *Lomatium triternatum* var. *triternatum*, on the other hand, cannot be defined chemically with the data available; there was too much variation in the essential oil compositions.

In the *Lomatium dissectum* complex, it is easy to distinguish *L. dissectum* from *L. multifidum*. *Lomatium dissectum* essential oils were dominated by octyl acetate (42.6% ± 3.4%) and decyl acetate (40.4% ± 4.8%), which were detected in only minute, if at all, quantities in the other *Lomatium* essential oils. In contrast, *L. multifidum* had significantly higher myrcene concentrations (30.7% ± 13.2%) in its essential oils.

The volatile phytochemistry of *L. papilioniferum* seems to depend on geographical location. Collections from both Idaho and Oregon showed relatively high concentrations of *p*-cymene and γ-terpinene. β-Phellandrene was significantly higher in the Oregon samples (13.4% ± 11.9%) than the Idaho samples (trace amounts only), and sedanenolide concentrations were significantly greater in *L. papilioniferum* from Oregon (6.18% ± 5.26), which was not observed in any of the Idaho samples. Conversely, the *L. papilioniferum* essential oils from Idaho were dominated by 2-methyl-5-(1,2,2-trimethylcyclopentyl)phenol (29.0% ± 2.9%), which was virtually absent in the other *Lomatium* essential oils.

### 2.9. Enantiomeric Distributions

Enantioselective GC-MS was carried out on the *Lomatium* essential oil samples to examine the distribution of chiral terpenoid components. The enantiomeric distributions are summarized in Table 10. There is variation in the enantiomeric distributions, both between species and within species. In order to assess the differences between the species and sampling sites, the enantiomeric distributions of (+)-α-pinene, (−)-camphene, (+)-sabinene, (+)-β-pinene, (+)-limonene, and (+)-linalool were analyzed by an ANOVA followed by Tukey’s test using Minitab^®^ 18 (Minitab Inc., State College, PA, USA). Differences at *p* < 0.05 were considered to be statistically significant. (Table 11).

The three taxa in the *Lomatium triternatum* complex (*L. anomalum*, *L. packardiae*, and *L. triternatum* var. *triternatum*) are distinguished by significantly different α-pinene, sabinene, β-pinene, and limonene enantiomeric distributions. The (+)-α-pinene and (+)-sabinene levels are significantly greater in *L. anomalum* than in *L. packardiae* or *L. triternatum triternatum*.

Furthermore, (+)-β-pinene is significantly lower in *L. triternatum triternatum* than either *L. anomalum* or *L. packardiae*, and (+)-limonene is much greater in *L. packardiae* than *L. anomalum* or *L. triternatum triternatum*. There are significant differences in the limonene enantiomeric distributions between the Oregon *L. papilioniferum* samples and the Idaho *L. papilioniferum* samples. Likewise, (+)-limonene is significantly greater in *L. dissectum* compared with *L. multifidum*.

## 3. Materials and Methods

### 3.1. Plant Collection and Identification

The *L. anomalum*, *L. packardiae*, and *L. triternatum* plant samples were identified by W.N. Setzer using published botanical descriptions [2,3,10] and comparison with herbarium samples from the New York Botanical Garden [31,32,33] and the Intermountain Region Herbarium Network [34]. *Lomatium papilioniferum* was identified by W.N. Setzer using published botanical descriptions [13] and by comparison with herbarium samples from the New York Botanical Garden [35]. *Lomatium dissectum* and *L. multifidum* were identified by W.N. Setzer using published botanical descriptions [14] and verified by comparison with herbarium samples [36,37]. *Lomatium nudicaule* was identified in the field by W.N. Setzer using a field guide [5] and verified using published botanical descriptions [38,39,40] and herbarium samples from the New York Botanical Garden [41]. Voucher specimens of each species were deposited with the herbarium of the University of Alabama in Huntsville, and voucher numbers are presented in Table 12.

### 3.2. Hydrodistillation

The fresh plant materials were stored frozen (–20 °C) until distillation. For each sample, the fresh/frozen aerial parts were chopped and hydrodistilled using a Likens-Nickerson apparatus [42,43,44] with continuous extraction of the distillate for four hours. The chopped plant material was placed in a 1000-mL flask with enough distilled water to cover the material. Dichloromethane (25 mL) was used in the receiving flask. Evaporation of the dichloromethane gave the essential oils, summarized in Table 12.

### 3.3. Gas Chromatographic Analysis

The essential oils of the aerial parts of *L. anomalum*, *L. dissectum*, *L. multifidum*, *L. nudicaule*, *L. packardiae*, *L. papilioniferum*, and *L. triternatum* var. *triternatum* were analyzed by gas chromatography–mass spectrometry (GC-MS), gas chromatography coupled with flame ionization detection (GC-FID), and chiral GC-MS as previously described [45]. Instrumental details are provided as supplementary material (Supplementary Table S1). Retention indices (RIs) were determined using a homologous series of *n*-alkanes using the linear formula of van den Dool and Kratz [46]. The essential oil components were identified by comparing their retention index values (within ten RI units) and their mass spectral fragmentation patterns (>80% similarity) with those reported in the Adams [47], FFNSC3 [48], NIST20 [49], and Satyal [50] databases. The compound percentages were calculated from raw peak areas without standardization. The individual enantiomers were determined using enantioselective GC-MS by comparison of MS fragmentation and RI values with authentic samples (Sigma-Aldrich, Milwaukee, WI, USA), which were compiled in our in-house database. Percentages of each enantiomer were calculated from raw peak integration.

### 3.4. Statistical Analyses

An agglomerative hierarchical cluster analysis (HCA) and principal component analysis (PCA) were carried out using XLSTAT v. 2018.1.1.62926 (Addinsoft, Paris, France). The HCA and PCA analyses were carried out using the percentages of the most abundant components (*Lomatium triternatum* complex: limonene, sabinene, β-phellandrene, α-pinene, (*Z*)-ligustilide, myrcene, β-pinene, cryptone, (*E*)-β-ocimene, carotol, γ-terpinene, terpinen-4-ol, and spathulenol; *Lomatium dissectum* complex: β-myrcene, decyl acetate, octyl acetate, (*E*)-β-ocimene, 1-decanol, limonene, α-bisabolol, β-phellandrene, unidentified (RI 1959), 2-methyloct-(3*E*)-en-5-yne, longifolene, (*Z*)-β-ocimene, *p*-cymene, camphene, bornyl acetate, α-eudesmol, γ-terpinene, terpinolene, 1-octanol, γ-eudesmol, globulol, agarospyryl acetate, and viridiflorene; *Lomatium grayi* complex: *p*-cymene, γ-terpinene, limonene + β-phellandrene, 2-methyl-5-(1,2,2-trimethylcyclopentyl)phenol, sedanenolide, myrcene, (*E*)-β-ocimene, (*Z*)-β-ocimene, cuparene, 3-butylphthalide, piperitone, longifolene, humulol, terpinolene, α-pinene, (*E*)-nerolidol, and decyl acetate) from this study in addition to compositions previously reported. Dissimilarity was used to determine clusters considering Euclidean distance, and Ward’s method was used to define agglomeration. The PCA, type correlation, was carried out to verify the chemical associations (clusters) from the HCA analysis. An analysis of variance was conducted by a one-way ANOVA followed by the Tukey test [51] using Minitab^®^ 18 (Minitab Inc., State College, PA, USA). Differences at *p* < 0.05 were considered to be statistically significant.

## 4. Conclusions

In this work, the essential oils of seven species of *Lomatium* (*L. anomalum*, *L. dissectum*, *L. multifidum*, *L. nudicaule*, *L. packardiae*, *L. papilioniferum*, and *L. triternatum* var. *triternatum*) from the intermountain western United States were obtained and analyzed by gas chromatographic methods. This work complements previously published essential oil analyses of *Lomatium* species. In addition, the enantiomeric distributions of chiral terpenoid components in this work serve to further characterize the *Lomatium* species. The three species in the *Lomatium triternatum* complex can be distinguished by their essential oil compositions. *Lomatium packardiae* essential oil can be characterized as a limonene-rich essential oil, and *L. anomalum* is a species rich in sabinene and α-pinene. The essential oils of *L. dissectum* and *L. multifidum*, members of the *Lomatium dissectum* complex, are readily discriminated based on essential oil composition. *Lomatium multifidum* essential oils were rich in myrcene while *L. dissectum* essential oils were dominated by octyl acetate and decyl acetate. *Lomatium papilioniferum* essential oils from western Idaho are readily characterized by high *p*-cymene and 2-methyl-5-(1,2,2-trimethylcyclopentyl)phenol concentrations. North-central Oregon *L. papilioniferum* essential oils were variable but may be tentatively classified as high in β-phellandrene and sedanenolide. There are not enough consistent data to properly characterize the chemotype(s) of *L. triternatum* var. *triternatum*. Because of the variation observed in the Oregon *L. papilioniferum* essential oils, additional collection and analyses are needed to confidently describe the chemotype(s) of that species, as well as the *L. grayi* complex in general. Additional sampling from other geographical locations would be helpful. The life cycle and timing of the sampling could affect the composition; additional sampling throughout the phenological stages of each species would provide important additional information.

## Figures and Tables

**Table 1:** Major components of *Lomatium* essential oils reported in the literature.

*Lomatium* Species	Collection Site	Plant Tissue	Major Components (>5%)	Ref.
*Lomatium brandegeei* J.F. Macbr.	Slate Peak, Washington	Aerial parts	α-pinene (9.2%), β-phellandrene (60.9%)	[20]
*Lomatium dasycarpum* (Torr. & A. Gray) J.M. Coult. & Rose	Trinity National Forest, California	Leaves and stems	3-methyl-2-buten-1-yl 3-methylbutyrate (22.3%), lavandulyl 2-methylbutyrate (16.9%), senkyunolide (9.8%)	[21]
*Lomatium dissectum* (Nutt.) Mathias & Constance var. *dissectum*	Six Rivers National Forest, California	Aerial parts	1-octanol (9.0%), octyl acetate (5.3%), palmitic acid (15.3%)	[22]
*Lomatium dissectum* var. *multifidum* (Nutt.) Mathias & Constance (syn. *Lomatium multifidum* (Nutt.) R.P. McNeill & Darrach)	San Bernardino National Forest, California	Aerial parts	(3*Z*)-hexenol (18.5%), myrcene (6.0%), palmitic acid (8.6%)	[22]
*Lomatium eastwoodiae* (J.M. Coult. & Rose) J.F. Macbr.	Black Ridge, Colorado	Aerial parts	α-pinene (6.2%), myrcene (5.1%), limonene + β-phellandrene (12.9%), (*E*)-β-caryophyllene (12.2%), germacrene D 95.2%)	[20]
*Lomatium foeniculaceum* subsp. *fimbriatum* W.L. Theob.	Inyo National Forest, California	Leaves and stems	(3*Z*)-hexenol (6.5%), limonene + β-phellandrene (6.8%), terpinolene (6.7%), germacrene D (15.9%), (*Z*)-ligustilide (13.1%)	[23]
*Lomatium graveolens* (S. Watson) J.M. Coult. & Rose	Provo Peak, Utah	Aerial parts	β-pinene (21.6%), limonene + β-phellandrene (33.2%), osthole (5.2%)	[20]
*Lomatium grayi* “new variety” (based on the reported collection site, this is probably *Lomatium papilioniferum* J.A. Alexander & Whaley) [13]	Elko County, Nevada	Aerial parts	limonene + β-phellandrene (17.7%), γ-terpinene (16.1%), senkyunolide (44.0%)	[24]
*Lomatium grayi* (J.M. Coult & Rose) J.M. Coult. & Rose var. *grayi*	Utah County, Utah	Aerial parts	myrcene (8.4%), limonene + β-phellandrene (27.2%), γ-terpinene (10.4%), senkyunolide (24.4%)	[24]
*Lomatium grayi* var. *depauperatum* (M.E. Jones) Mathias	Juab County, Utah	Aerial parts	myrcene (8.1%), limonene + β-phellandrene (20.8%), (*Z*)-β-ocimene (18.9%), (*Z*)-ligustilide (6.7%)	[24]
*Lomatium howelii* (S. Watson) Jeps.	Eight Dollar Mountain, Oregon	Aerial parts	(*E*)-β-ocimene (5.8%), octyl acetate (24.8%), citronellyl acetate (7.1%), decyl acetate (6.7%), lauryl acetate (5.1%)	[20]
*Lomatium howelii* (S. Watson) Jeps.	Low Divide, California	Aerial parts	1-octanol (11.4%), octyl acetate (23.5%), citronellyl acetate (6.0%), germacrene D (6.0%)	[20]
*Lomatium junceum* Barneby & N.H. Holmgren	Emery County, Utah	Aerial parts	α-pinene (24.3%), β-pinene (29.3%), limonene + β-phellandrene (11.3%)	[20]
*Lomatium lucidum* Jeps.	San Bernardino National Forest, California	Leaves and stems	limonene + β-phellandrene (11.5%), decanal (15.7%), bornyl/isobornyl acetate (6.1%), dodecanal (9.4%), α-humulene	[21]
*Lomatium macrocarpum* J.M. Coult. & Rose	Six Rivers National Forest, California	Leaves and stems	(3*Z*)-hexenol (9.2%), (*E*)-β-caryophyllene (12.6%), palmitic acid (9.0%), linoleic acid (5.2%)	[21]
*Lomatium marginatum* var. *purpureum* (Jeps.) Jeps.	Lake County, California	Leaves and stems	(3*Z*)-hexenol (10.3%), (*Z*)-β-lomatene (12.9%), (*E*)-β-caryophyllene (9.3%)	[25]
*Lomatium mohavense* (J.M. Coult. & Rose) J.M. Coult. & Rose subsp. *mohavense*	Grant, California	Leaves and stems	limonene + β-phellandrene (6.0%), *trans*-β-elemene (17.8%), (*E*)-β-caryophyllene (7.8%), germacrene D (10.8%), bicyclogermacrene (6.2%)	[26]
*Lomatium mohavense* subsp. *longilobum* W.L. Theob.	Acton, California	Leaves and stems	(3*Z*)-hexenol (7.5%), limonene + β-phellandrene (6.5%), β-sinensal (6.8%), *iso*-α-sinensal (19.3%), α-sinensal (5.4%), *iso*-α-sinensyl acetate (5.7%)	[26]
*Lomatium nevadense* (S. Watson) J.M. Coult. & Rose var. *parishii* (J.M. Coult. & Rose) Jeps.	Bishop, California	Leaves and stems	(*E*)-β-ocimene (5.1%), (*E*)-β-caryophyllene (10.3%), germacrene D (10.7%), bicyclogermacrene (7.0%),	[27]
*Lomatium parryi* (S. Watson) J.F. Macbr.	Pine Valley Mountains, Utah	Aerial parts	limonene + β-phellandrene (12.8%), bornyl acetate (18.6%)	[20]
*Lomatium rigidum* (M.E. Jones) Jeps.	Eastern Sierra Nevada Mountains, California	Leaves and stems	limonene + β-phellandrene (9.1%), δ-cadinene (12.4%), τ-cadinol + τ-muurolol (9.0%), α-cadinol (16.4%), (*Z*)-falcarinol (10.8%)	[28]
*Lomatium rigidum* (M.E. Jones) Jeps.	Bishop Canyon, California	Aerial parts	α-pinene (6.9%), limonene + β-phellandrene (28.6%), cryptone (5.6%), osthole (10.9%)	[29]
*Lomatium scabrum* (J.M. Coult. & Rose) Mathias var. *tripinnatum* Goodrich	St. George, Utah	Aerial parts	myrcene (8.2%), limonene + β-phellandrene (26.0%), (*Z*)-β-ocimene (11.8%)	[29]
*Lomatium torreyi* (J.M. Coult. & Rose) J.M. Coult. & Rose	Yosemite National Park, California	Aerial parts	β-phellandrene (12.9%), (*Z*)-β-ocimene (14.2%), (*Z*)-ligustilide (42.4%)	[30]
*Lomatium utriculatum* (Nutt. ex Torr. & A. Gray) J.M. Coult. & Rose	Six Rivers National Forest, California	Leaves and stems	(*Z*)-ligustilide (19.6%), palmitic acid (15.3%)	[21]

**Table 2:** Essential oil composition (%) of *Lomatium anomalum* Jones ex J.M. Coult. & Rose.

RI_calc_	RI_db_	Compound	La#1	La#2	La#3
800	801	Hexanal	tr	tr	tr
832	831	Furfural	-	-	tr
850	849	(2*E*)-Hexenal	0.1	-	tr
853	853	(3Z)-Hexenol	tr	-	tr
903	906	Heptanal	-	tr	tr
926	927	α-Thujene	0.3	0.8	0.7
934	933	α-Pinene	37.6	21.9	23.6
950	950	Camphene	0.1	0.1	0.1
975	972	Sabinene	48.2	48.0	49.9
980	978	β-Pinene	2.2	3.2	3.7
990	989	Myrcene	0.3	0.9	1.4
992	990	Dehydro-1,8-cineole	tr	tr	tr
1005	1004	*p*-Mentha-1(7),8-diene	tr	tr	tr
1007	1007	α-Phellandrene	tr	tr	tr
1010	1009	δ-3-Carene	-	tr	tr
1017	1018	α-Terpinene	0.5	1.5	1.3
1025	1025	*p*-Cymene	0.3	0.8	0.4
1030	1030	Limonene	0.7	1.7	1.2
1031	1031	β-Phellandrene	0.9	1.8	2.0
1032	1032	1,8-Cineole	-	-	tr
1034	1034	(*Z*)-β-Ocimene	tr	tr	tr
1043	1045	Phenylacetaldehyde	-	tr	tr
1045	1045	(*E*)-β-Ocimene	0.1	0.6	0.7
1058	1058	γ-Terpinene	2.7	7.0	4.7
1070	1069	*cis*-Sabinene hydrate	0.4	0.7	0.7
1072	1071	Dehydromyrcenol	-	-	tr
1086	1086	Terpinolene	0.5	1.3	0.9
1091	1093	*p*-Cymenene	-	tr	tr
1097	1099	6-Camphenone	tr	tr	tr
1100	1101	Linalool	0.1	tr	0.1
1101	1101	*trans*-Sabinene hydrate	0.4	0.6	0.6
1105	1107	Nonanal	-	-	tr
1107	1107	1-Octen-3-yl acetate	-	-	tr
1113	1113	*p*-Mentha-1,3,8-triene	-	0.1	tr
1114	1112	(*E*)-2,4-Dimethylhepta-2,4-dienal	-	-	tr
1121	1122	*trans-p*-Mentha-2,8-dien-1-ol	-	-	tr
1125	1124	*cis-p*-Menth-2-en-1-ol	0.2	0.3	0.3
1135	1135	2-Vinylanisole	-	0.1	tr
1143	1142	*trans*-*p*-Menth-2-en-1-ol	0.1	0.2	0.2
1146	1145	*trans*-Verbenol	tr	tr	tr
1150	1152	1,4-Dimethyl-4-acetylcyclohexene	tr	0.1	tr
1179	1179	2-Isopropenyl-5-methyl-4-hexenal	tr	tr	tr
1181	1180	Terpinen-4-ol	2.6	5.1	4.8
1187	1186	*p*-Cymen-8-ol	tr	0.1	tr
1195	1195	α-Terpineol	0.2	0.2	0.2
1197	1196	*cis*-Piperitol	tr	tr	tr
1210	1209	*trans*-Piperitol	tr	tr	tr
1224	1231	*trans*-Chrysenthenyl acetate	0.1	0.2	0.1
1240	1240	Ascaridole	-	-	0.1
1305	1306	*iso*-Ascaridole	-	-	0.1
1309	1309	*p*-Vinylguaiacol	tr	tr	tr
1351	1357	Eugenol	0.1	-	-
1376	1377	α-Copaene	tr	tr	tr
1384	1382	β-Bourbonene	-	tr	tr
1390	1390	*trans*-β-Elemene	-	tr	tr
1400	1403	Methyl eugenol	tr	tr	-
1411	1412	Longifolene	tr	0.3	0.1
1420	1417	(*E*)-β-Caryophyllene	0.2	0.1	0.1
1431	1432	γ-Elemene	tr	tr	0.1
1433	1432	*trans*-α-Bergamotene	0.1	0.1	0.1
1453	1452	(*E*)-β-Farnesene	-	tr	tr
1456	1454	α-Humulene	tr	tr	tr
1481	1480	Germacrene D	0.2	0.2	0.3
1496	1497	Bicyclogermacrene	0.1	0.3	0.2
1518	1518	δ-Cadinene	tr	tr	tr
1559	1557	Germacrene B	tr	0.1	0.1
1561	1560	(*E*)-Nerolidol	0.1	0.1	tr
1577	1576	Spathulenol	-	tr	tr
1603	1601	Carotol	-	0.1	-
1669	1669	(2*E*,6*Z*)-Farnesol	0.1	tr	-
1728	1730	(*Z*)-Ligustilide	-	0.8	0.5
1779	1776	2-Methyl-5-(1,2,2-trimethylcyclopentyl)phenol	-	-	0.1
1788	1790	(*E*)-Ligustilide	-	tr	tr
1842	1841	Phytone	-	tr	tr
2149	2143	Serratol	-	0.1	-
2300	2300	Tricosane	0.1	tr	tr
2400	2400	Tetracosane	-	-	0.5
2500	2500	Pentacosane	0.1	0.1	tr
2575	2595	Selinidin	0.3	-	-
2700	2700	Heptacosane	0.2	0.2	0.1
		Monoterpene hydrocarbons	94.3	89.9	90.6
		Oxygenated monoterpenoids	3.9	7.4	7.2
		Sesquiterpene hydrocarbons	0.6	1.0	0.9
		Oxygenated sesquiterpenoids	0.1	0.2	0.1
		Diterpenoids	0.0	0.1	0.0
		Benzenoid aromatics	0.4	0.1	0.0
		Others	0.5	1.0	1.1
		Total identified	99.9	99.7	100.0

RI_calc_ = retention index calculated with respect to a homologous series of *n*-alkanes on a ZB-5ms column. RI_db_ = reference retention index values obtained from the databases. La = *Lomatium anomalum*. tr = trace (<0.05%). - = not observed.

**Table 3:** Essential oil composition (%) of *Lomatium packardiae* Cronguist.

RI_calc_	RI_db_	Compounds	Lpack#1	Lpack#2	Lpack#3	Lpack#4
925	925	α-Thujene	tr	tr	0.1	0.1
933	932	α-Pinene	0.5	0.4	1.5	1.5
949	950	Camphene	tr	tr	tr	tr
972	971	Sabinene	0.1	0.1	2.3	0.4
977	978	β-Pinene	1.6	0.6	2.1	2.3
989	989	Myrcene	2.4	3.1	3.6	3.1
990	990	Dehydro-1,8-cineole	-	-	tr	tr
1005	1004	*p*-Mentha-1(7),8-diene	0.1	0.1	tr	0.1
1007	1006	α-Phellandrene	0.1	0.7	0.2	0.4
1009	1008	δ-3-Carene	tr	tr	tr	tr
1017	1018	α-Terpinene	tr	tr	tr	tr
1025	1025	*p*-Cymene	0.1	0.1	0.1	tr
1030	1030	Limonene	72.2	65.0	48.6	57.8
1031	1031	β-Phellandrene	4.4	6.2	5.1	5.4
1035	1034	(*Z*)-β-Ocimene	-	-	0.1	0.1
1044	1045	Phenylacetaldehyde	tr	tr	tr	tr
1045	1045	(*E*)-β-Ocimene	0.1	0.1	2.1	1.7
1057	1057	γ-Terpinene	tr	tr	0.3	tr
1070	1069	*cis*-Sabinene hydrate	tr	tr	tr	tr
1071	1071	Dihydromyrcenol	tr	tr	-	-
1085	1086	Terpinolene	tr	tr	tr	tr
1090	1090	6,7-Epoxymyrcene	tr	tr	-	-
1098	1098	Perillene	tr	tr	-	-
1099	1101	Linalool	tr	tr	tr	tr
1101	1101	*trans*-Sabinene hydrate	tr	tr	tr	tr
1105	1104	Nonanal	tr	tr	tr	tr
1122	1122	*trans*-*p*-Mentha-2,8-dien-1-ol	0.1	tr	tr	tr
1125	1124	*cis*-*p*-Menth-2-en-1-ol	tr	0.1	0.1	0.1
1127	1127	α-Campholenal	tr	-	-	-
1131	1131	Limona ketone	tr	tr	-	-
1133	1134	*cis*-Limonene oxide	0.2	tr	tr	tr
1136	1137	*cis*-*p*-Mentha-2,8-dien-1-ol	tr	tr	-	-
1137	1137	*trans*-Limonene oxide	0.1	tr	tr	tr
1142	1142	*trans*-*p*-Menth-2-en-1-ol	tr	tr	tr	tr
1145	1146	Oxophorone	-	-	0.1	tr
1156	1156	Pentylbenzene	tr	tr	-	tr
1158	1161	Pentylcyclohexa-1,3-diene	0.1	0.2	0.1	0.2
1180	1180	Terpinen-4-ol	tr	tr	0.2	tr
1187	1187	Cryptone	0.3	0.1	tr	tr
1195	1195	α-Terpineol	tr	tr	tr	tr
1197	1198	*cis*-Piperitol	-	-	tr	tr
1203	1202	*cis*-Sabinol	tr	tr	-	-
1218	1218	*trans*-Carveol	tr	tr	-	-
1242	1242	Cuminal	tr	-	-	-
1243	1246	Carvone	tr	tr	-	-
1265	1265	Dec-(2*E*)-enal	tr	tr	-	-
1277	1277	Phellandral	tr	tr	-	-
1286	1286	α-Terpinen-7-al	tr	tr	-	-
1288	1286	*trans*-Sabinyl acetate	-	-	0.1	-
1338	1339	3-Oxo-*p*-menth-1-en-7-al	tr	tr	-	-
1378	1380	Daucene	tr	tr	0.2	0.2
1388	1390	*trans*-β-Elemene	-	tr	0.1	tr
1410	1410	Dodecanal	-	-	tr	tr
1417	1417	(*E*)-β-Caryophyllene	tr	tr	0.1	tr
1417	1416	β-Funebrene	-	tr	tr	tr
1429	1430	γ-Elemene	tr	tr	0.1	tr
1433	1433	*trans*-α-Bergamotene	-	-	tr	tr
1452	1452	(*E*)-β-Farnesene	0.1	0.1	0.3	0.2
1454	1454	α-Humulene	-	-	tr	tr
1472	1473	Dauca-5,8-diene	-	-	0.2	-
1475	1475	γ-Muurolene	-	-	-	0.2
1480	1480	Germacrene D	0.1	0.2	1.4	0.7
1494	1494	α-Zingiberene	tr	0.1	0.1	0.1
1495	1497	Bicyclogermacrene	-	-	0.3	tr
1501	1504	*iso*-Daucene	-	-	tr	tr
1507	1508	β-Bisabolene	-	-	tr	tr
1511	1512	α-Alaskene	-	tr	tr	tr
1513	1512	γ-Cadinene	-	-	tr	-
1518	1518	δ-Cadinene	-	-	tr	tr
1523	1523	β-Sesquiphellandrene	0.5	1.0	1.1	0.9
1557	1557	Germacrene B	tr	0.1	0.1	0.1
1576	1574	Germacra-1(10),5-dien-4β-ol	-	-	-	tr
1577	1576	Spathulenol	tr	-	0.1	-
1581	1584	10-*epi*-Juneol	tr	tr	tr	tr
1582	1587	Caryophyllene oxide	-	-	-	-
1601	1601	Carotol	2.2	0.2	7.0	7.2
1612	1615	Zingiberenol	tr	tr	tr	tr
1613	1613	Tetradecanal	-	tr	tr	tr
1649	1649	3-Butylphthalide	-	-	-	0.1
1655	1655	α-Cadinol	-	-	tr	tr
1662	1664	*ar*-Turmerone	tr	-	0.1	-
1668	1669	(3*Z*)-Butylidene phthalide	0.6	0.3	0.1	0.2
1668	1668	α-Turmerone	-	-	0.5	-
1687	1687	Himachal-4-en-1β-ol	-	0.1	0.1	0.1
1700	1699	Curlone B (=β-Turmerone)	tr	-	0.2	-
1712	1712	Senkyunolide (=Sedanenolide)	-	-	-	0.1
1712	1719	(3*E*)-Butylidene phthalide	0.2	0.2	0.2	0.1
1729	1730	(*Z*)-Ligustilide	12.3	18.0	19.1	15.4
1781	1776	2-Methyl-5-(1,2,2-trimethylcyclopentyl)phenol	-	0.1	tr	0.1
1788	1790	(*E*)-Ligustilide	1.0	2.6	1.2	1.1
2038	2037	(*Z*)-Falcarinol	-	tr	0.1	0.2
2300	2300	Tricosane	tr	tr	tr	tr
2400	2400	Tetracosane	tr	tr	tr	tr
2500	2500	Pentacosane	0.1	0.1	0.3	0.1
2600	2600	Hexacosane	-	-	tr	tr
2700	2700	Heptacosane	tr	tr	0.2	tr
		Monoterpene hydrocarbons	81.5	76.4	66.1	72.9
		Oxygenated monoterpenoids	0.7	0.2	0.5	0.1
		Sesquiterpene hydrocarbons	0.7	1.4	4.0	2.3
		Oxygenated sesquiterpenoids	2.2	0.3	7.8	7.3
		Benzenoid aromatics	14.1	21.2	20.5	16.9
		Others	0.1	0.3	0.7	0.5
		Total identified	99.2	99.8	99.7	100.0

RI_calc_ = retention index calculated with respect to a homologous series of *n*-alkanes on a ZB-5ms column. RI_db_ = reference retention index values obtained from the databases. Lpack = *Lomatium packardiae*. tr = trace (<0.05%). - = not observed.

**Table 4:** Essential oil composition (%) of *Lomatium triternatum* (Pursh) J.M. Coult. & Rose var. *triternatum*.

RI_calc_	RI_db_	Compounds	Ltt#1	Ltt#2	Ltt#3
925	925	α-Thujene	0.1	0.2	0.6
933	932	α-Pinene	0.6	5.7	9.1
949	950	Camphene	tr	0.3	0.2
972	971	Sabinene	5.6	2.1	9.9
977	978	β-Pinene	0.5	10.6	11.9
989	989	Myrcene	12.7	2.9	14.1
990	990	Dehydro-1,8-cineole	0.1	0.4	0.2
1005	1004	*p*-Mentha-1(7),8-diene	0.4	0.5	0.2
1007	1006	α-Phellandrene	0.2	-	0.4
1009	1008	δ-3-Carene	0.1	-	tr
1017	1018	α-Terpinene	-	-	0.1
1019	1024	2-Cyclohexene-1,4-dione	-	0.6	-
1025	1025	*p*-Cymene	2.1	4.3	0.7
1030	1030	Limonene	1.6	4.7	1.1
1031	1031	β-Phellandrene	48.5	1.7	29.4
1035	1034	(*Z*)-β-Ocimene	0.5	-	0.5
1045	1045	(*E*)-β-Ocimene	8.2	-	9.2
1057	1057	γ-Terpinene	0.3	-	0.3
1070	1069	*cis*-Sabinene hydrate	0.1	0.3	0.1
1071	1071	Dihydromyrcenol	0.2	0.6	tr
1085	1086	Terpinolene	-	-	0.1
1090	1090	6,7-Epoxymyrcene	0.1	0.4	0.1
1091	1091	Rosefuran	0.1	-	-
1095	1097	α-Pinene oxide	0.1	-	tr
1098	1098	Perillene	tr	0.2	-
1099	1101	Linalool	0.3	1.3	0.1
1101	1101	*trans*-Sabinene hydrate	0.1	0.1	0.1
1105	1104	Nonanal	tr	0.2	tr
1107	1109	1-Octen-3-yl acetate	0.1	0.2	-
1125	1124	*cis*-*p*-Menth-2-en-1-ol	0.2	0.8	0.1
1127	1127	α-Campholenal	-	0.3	-
1129	1129	(*Z*)-Myroxide	tr	-	tr
1131	1131	Limona ketone	-	-	-
1133	1134	*cis*-Limonene oxide	-	0.4	-
1138	1138	Benzeneacetonitrile	0.1	-	tr
1139	1139	(*E*)-Myroxide	0.2	-	0.1
1139	1139	Nopinone	-	0.5	-
1141	1141	*trans*-Pinocarveol	-	0.6	-
1142	1142	*trans*-*p*-Menth-2-en-1-ol	0.1	0.5	0.1
1145	1146	Oxophorone	0.1	-	tr
1146	1146	*trans*-Verbenol	-	0.2	tr
1162	1164	Pinocarvone	-	0.6	-
1169	1169	Rosefuran epoxide	0.1	-	-
1180	1180	Terpinen-4-ol	0.5	0.8	0.7
1187	1187	Cryptone	3.7	17.9	0.8
1192	1192	Methyl salicylate	0.1	-	-
1195	1195	α-Terpineol	0.2	0.6	0.2
1196	1196	Myrtenal	-	0.8	-
1197	1195	*p*-Menth-3-en-7-al	0.2	0.8	0.1
1197	1198	*cis*-Piperitol	-	-	-
1203	1202	*cis*-Sabinol	0.1	-	0.1
1223	1223	*m*-Cumenol	-	0.5	-
1242	1242	Cuminal	0.3	2.8	0.1
1243	1246	Carvone	-	0.3	-
1254	1254	Piperitone	0.1	0.4	-
1264	1258	*trans*-Piperitone epoxide	0.2	0.5	-
1265	1265	Dec-(2*E*)-enal	-	0.5	-
1286	1286	α-Terpinen-7-al	0.1	0.2	tr
1291	1291	*p*-Cymen-7-ol	0.3	2.4	0.1
1322	1318	4-Hydroxycryptone	-	1.5	-
1331	1330	Bicycloelemene	-	-	0.1
1338	1339	3-Oxo-*p*-menth-1-en-7-al	0.4	2.4	0.1
1388	1390	*trans*-β-Elemene	-	-	0.1
1417	1417	(*E*)-β-Caryophyllene	0.1	-	1.1
1442	---	Unidentified ^a^	0.5	3.5	0.1
1448	---	Unidentified ^b^	-	1.2	-
1454	1454	α-Humulene	-	-	0.1
1475	1475	γ-Muurolene	0.2	-	0.1
1480	1480	Germacrene D	3.0	-	2.5
1491	---	Unidentified ^c^	0.5	2.5	0.1
1495	1497	Bicyclogermacrene	-	-	0.8
1507	1508	β-Bisabolene	0.1	-	-
1513	1512	γ-Cadinene	0.1	-	tr
1518	1518	δ-Cadinene	0.2	-	0.1
1576	1574	Germacra-1(10),5-dien-4β-ol	0.3	-	0.2
1577	1576	Spathulenol	-	6.3	0.3
1582	1587	Caryophyllene oxide	-	1.2	0.1
1632	1630	Caryophylla-4(12),8(13)-dien-5α-ol	-	-	0.1
1639	1644	*allo*-Aromadendrene epoxide	0.3	-	0.1
1642	1642	τ-Cadinol	-	-	tr
1643	1644	τ-Muurolol	-	-	0.1
1649	1649	3-Butylphthalide	-	0.4	-
1655	1655	α-Cadinol	0.3	0.4	0.2
1662	1664	*ar*-Turmerone	0.2	0.3	0.1
1693	1686	Shyobunol	0.1	-	-
1712	1712	Senkyunolide (=Sedanenolide)	0.4	-	0.2
1806	1807	Tetradecyl acetate	-	-	0.1
1872	1875	Oplopanonyl acetate	0.1	0.5	0.1
1936	1933	Beyerene	0.2	0.7	0.1
2038	2037	(*Z*)-Falcarinol	0.1	-	0.1
2300	2300	Tricosane	-	-	0.1
2400	2400	Tetracosane	-	-	0.9
2500	2500	Pentacosane	0.1	0.3	0.2
2612	2610	Auraptene	1.0	1.3	0.9
2700	2700	Heptacosane	0.3	0.7	0.4
		Monoterpene hydrocarbons	81.6	33.0	87.9
		Oxygenated monoterpenoids	8.0	39.1	2.8
		Sesquiterpene hydrocarbons	3.6	0.0	4.9
		Oxygenated sesquiterpenoids	1.3	8.8	1.1
		Diterpenoids	0.2	0.7	0.1
		Benzenoid aromatics	1.1	1.7	0.9
		Others	1.0	2.5	2.0
		Total identified	96.8	85.6	99.7

RI_calc_ = retention index calculated with respect to a homologous series of *n*-alkanes on a ZB-5ms column. RI_db_ = reference retention index values obtained from the databases. Ltt = *Lomatium triternatum* var. *triternatum*. tr = trace (<0.05%). - = not observed. ^a^ MS(EI): 168 (25%), 139 (43%), 125 (86%), 107 (20%), 97 (77%), 79 (54%), 69 (73%), 55 (66%), 41 (76%), 41 (100%). ^b^ MS(EI): 139 (100%), 121 (48%), 109 (21%), 95 (37%), 92 (43%), 91 (38%), 83 (47%), 81 (49%), 19 (46%), 69 (54%), 55 (39%), 43 (46%), 41 (48%). ^c^ MS(EI): 152 (7%), 139 (18%), 110 (25%), 100 (20%), 82 (69%), 81 (100%), 71 (33%), 55 (27%), 43 (35%), 41 (41%).

**Table 5:** Essential oil compositions (%) of *Lomatium dissectum* (Nutt.) Mathias & Constance.

RI_calc_	RI_db_	Compounds	Ld#1	Ld#2	Ld#3	Ld#4	Ld#5
783	782	Prenol	0.1	tr	0.1	0.1	0.1
933	933	α-Pinene	tr	tr	tr	0.1	tr
950	950	Camphene	tr	tr	tr	tr	tr
973	972	Sabinene	tr	tr	tr	tr	tr
979	978	β-Pinene	tr	tr	tr	0.3	tr
990	991	Myrcene	tr	tr	tr	tr	tr
992	990	Dehydro-1,8-cineole	tr	tr	tr	tr	tr
1004	1006	Octanal	tr	tr	tr	tr	tr
1005	1005	(3*Z*)-Hexenyl acetate	tr	tr	tr	tr	tr
1007	1006	α-Phellandrene	tr	tr	-	tr	tr
1012	1012	Hexyl acetate	tr	tr	0.1	0.1	0.1
1025	1025	*p*-Cymene	tr	tr	tr	tr	tr
1029	1030	Limonene	tr	tr	tr	tr	tr
1031	1031	β-Phellandrene	tr	tr	tr	tr	tr
1033	1032	1,8-Cineole	tr	tr	tr	tr	tr
1035	1034	(*Z*)-β-Ocimene	-	-	tr	-	tr
1044	1045	Phenylacetaldehyde	tr	tr	tr	tr	tr
1046	1046	(*E*)-β-Ocimene	tr	tr	tr	tr	tr
1058	1057	γ-Terpinene	tr	tr	-	tr	-
1070	1069	1-Octanol	0.6	0.3	1.1	0.8	0.6
1086	1087	Terpinolene	-	-	-	-	tr
1092	1093	2-Nonanone	-	-	-	tr	-
1100	1101	Linalool	tr	tr	tr	tr	tr
1105	1107	Nonanal	tr	tr	tr	tr	tr
1108	1107	1-Octen-3-yl acetate	tr	tr	-	tr	tr
1124	1123	Methyl octanoate	tr	tr	tr	tr	tr
1143	1142	Epoxyterpinolene	tr	-	-	-	tr
1151	1152	1,4-Dimethyl-4-acetylcyclohexene	tr	-	-	-	tr
1152	1152	Nerol oxide	tr	-	-	-	-
1158	1160	Pentylcyclohexa-1,3-diene	-	tr	-	-	-
1179	1179	2-isopropenyl-5-methyl-4-hexenal	tr	-	-	-	tr
1181	1180	Terpinen-4-ol	tr	tr	-	-	-
1189	1189	*p*-Cymen-8-ol	-	tr	-	-	-
1196	1195	α-Terpineol	tr	tr	tr	tr	tr
1200	1202	(2*Z*)-Octenyl acetate	tr	-	tr	-	-
1207	1208	Decanal	0.7	0.3	0.4	0.2	0.3
1211	1211	Octyl acetate	41.1	43.3	48.4	42.4	37.8
1216	1217	Coumaran	-	tr	tr	tr	tr
1225	1231	*trans*-Chrysanthenyl acetate	tr	tr	-	-	tr
1255	1257	2-Phenethyl acetate	-	-	-	-	tr
1263	1263	(2*E*)-Decenal	-	-	-	tr	tr
1273	1271	1-Decanol	18.4	12.2	14.5	9.8	13.3
1284	1284	Lavandulyl acetate	tr	-	-	-	-
1293	1293	2-Undecanone	-	-	-	tr	tr
1310	1309	1-Nonyl acetate	tr	tr	tr	tr	0.1
1312	1310	*trans*-Ocimenyl acetate	-	-	-	-	tr
1359	1361	Neryl acetate	0.1	tr	tr	-	tr
1365	1367	Decanoic acid	tr	-	-	-	-
1376	1375	α-Copaene	-	-	tr	-	-
1379	1379	(*E*)-β-Damascenone	-	-	tr	-	-
1387	1384	(5*E*)-Decen-1-yl acetate	tr	0.1	tr	tr	tr
1388	1388	(3*Z*)-Decen-1-yl acetate	tr	0.1	tr	tr	0.1
1409	1408	Decyl acetate	37.2	42.0	33.9	43.2	45.8
1414	1414	Acora-3,7(14)-diene	-	-	-	tr	-
1419	1417	(*E*)-β-Caryophyllene	0.1	tr	0.1	0.1	0.1
1427	1428	β-Duprezianene	-	-	-	tr	-
1452	1452	(*E*)-β-Farnesene	-	-	-	tr	-
1456	1454	α-Humulene	tr	-	tr	tr	tr
1474	1476	1-Dodecanol	1.3	1.0	1.0	0.7	1.3
1488	1489	β-Selinene	-	-	-	tr	-
1490	1489	(*Z*,*E*)-α-Farnesene	-	tr	tr	-	tr
1494	1494	2-Tridecanone	-	-	-	tr	tr
1495	1497	α-Selinene	-	-	-	tr	-
1504	1504	(*E*,*E*)-α-Farnesene	-	tr	tr	tr	tr
1508	1507	1-Pentadecene	tr	tr	-	-	tr
1511	1512	α-Alaskene	-	tr	-	tr	-
1518	1518	δ-Cadinene	-	-	tr	-	-
1523	1523	β-Sesquiphellandrene	-	tr	-	tr	-
1529	1528	Kessane	-	tr	-	-	-
1560	1561	(*E*)-Nerolidol	tr	tr	tr	tr	tr
1582	1582	Octyl hexanoate	tr	tr	tr	0.1	tr
1602	1601	Carotol	-	tr	tr	-	-
1608	1607	1-Dodecyl acetate	0.3	0.3	0.2	0.4	0.4
1655	1655	α-Cadinol	-	-	tr	tr	-
1685	1686	*epi*-α-Bisabolol	-	-	-	tr	-
1704	1699	β-Cedr-8-en-15-ol	-	-	-	1.5	-
1720	1722	3-Isobutylidene phthalide	-	tr	tr	tr	-
1727	1730	(*Z*)-Ligustilide	-	0.1	tr	-	tr
1777	1779	Octyl octanoate	tr	tr	tr	0.1	tr
1779	1780	(*Z*)-Nerolidyl isobutyrate	tr	tr	-	-	-
1958	1958	Palmitic acid	tr	-	tr	-	-
1975	1978	Decyl octanoate	-	tr	tr	tr	tr
2046	2050	Bergaptene	tr	tr	tr	tr	tr
2148	2149	Incensyl acetate	tr	tr	tr	tr	-
2198	2192	Geranylgeraniol	tr	-	-	-	-
2301	2300	Tricosane	tr	tr	tr	tr	tr
2501	2500	Pentacosane	tr	tr	tr	tr	tr
2700	2700	Heptacosane	tr	tr	tr	tr	tr
		Isoprenoids	0.2	0.0	0.2	2.1	0.2
		Benzenoid aromatics	trace	0.1	trace	trace	trace
		Fatty acid derivatives	99.6	99.7	99.7	97.8	99.6
		Others	0.0	trace	0.0	0.0	0.0
		Total identified	99.8	99.7	99.8	99.9	99.8

RI_calc_ = retention index calculated with respect to a homologous series of *n*-alkanes on a ZB-5ms column. RI_db_ = reference retention index values obtained from the databases. Ld = *Lomatium dissectum*. tr = trace (<0.05%). - = not observed.

**Table 6:** Chemical compositions (%) of the essential oils of *Lomatium multifidum* (Nutt.) R.P. McNeill & Darrach.

RI_calc_	RI_db_	Compound	Lm#1 (OR)	Lm#2 (OR)	Lm#3 (OR)	Lm#4 (OR)	Lm#5 (OR)	Lm#6 (OR)	Lm#7 (ID)	Lm#8 (ID)	Lm#9 (ID)	Lm#10 (OR)	Lm#11 (OR)	Lm#12 (OR)
781	782	3-Methylbut-2-en-1-ol	-	-	1.7	1.4	1.7	-	-	-	-	2.0	2.6	2.2
790	790	3-Methyl-2-butenal	-	-	0.2	0.1	-	-	-	-	-	-	-	0.2
801	802	Hexanal	-	-	-	-	-	-	-	-	-	tr	0.1	0.1
850	850	(2*E*)-Hexenal	0.3	0.3	0.2	0.2	-	-	-	-	-	0.1	0.8	0.4
852	853	(3*Z*)-Hexenol	-	-	-	-	-	-	-	-	-	-	0.1	-
903	905	Heptanal	-	-	0.1	0.2	-	0.1	0.3	tr	0.1	-	0.1	0.1
920	921	Hashishene	tr	0.1	0.3	0.2	0.1	0.1	0.1	tr	tr	0.1	0.1	0.2
922	923	Tricyclene	tr	tr	0.1	tr	-	-	-	-	-	tr	tr	tr
933	933	α-Pinene	0.6	0.3	0.3	0.2	0.4	-	tr	0.1	0.3	0.3	1.2	1.4
947	948	α-Fenchene	tr	tr	tr	tr	tr	tr	tr	tr	tr	tr	tr	0.1
949	950	Camphene	4.2	1.7	4.8	2.0	0.9	0.4	tr	0.5	0.5	0.5	3.7	2.5
952	955	Propylbenzene	-	0.1	1.0	0.8	0.5	-	3.5	tr	-	-	0.3	0.4
965	963	2-Methyl-(3E)-octen-5-yne	0.1	0.3	0.6	0.2	0.1	7.6	6.9	5.5	7.8	tr	-	0.1
972	972	Sabinene	-	-	-	-	-	-	-	-	-	0.1	0.4	0.1
975	981	α-Myrcene	-	0.1	-	-	-	tr	-	-	-	-	-	-
978	978	β-Pinene	-	-	-	-	-	-	-	-	-	0.1	0.1	0.4
989	991	β-Myrcene	46.7	54.1	38.9	31.2	12.5	37.5	33.2	18.2	23.8	12.9	21.1	38.0
991	992	1,5,5-Trimethyl-3-methylene-1-cyclohexene	-	0.1	-	-	-	-	-	-	-	-	-	-
991	990	Dehydro-1,8-cineole	-	-	-	-	-	-	-	-	-	-	0.2	-
992	986	*cis-m*-Mentha-2,8-diene	-	-	-	-	-	0.1	-	-	-	-	-	-
1004	1004	*p*-Mentha-1(7),8-diene	-	-	-	-	-	-	-	-	-	0.1	0.2	-
1007	1007	α-Phellandrene	-	-	-	tr	0.3	-	-	-	-	1.0	tr	-
1024	1025	*p*-Cymene	0.7	1.0	1.1	0.3	14.8	0.2	0.1	0.2	0.8	1.6	2.8	0.5
1029	1030	Limonene	3.3	2.8	4.5	1.8	8.8	1.7	0.7	5.2	14.0	1.3	3.8	2.8
1030	1031	β-Phellandrene	0.3	0.2	0.1	2.5	4.1	0.1	0.1	tr	0.1	19.6	21.3	0.2
1032	1032	1,8-Cineole	-	-	-	-	-	-	-	-	-	0.1	tr	tr
1034	1033	Benzyl alcohol	-	-	-	-	-	0.1	-	-	-	-	-	-
1034	1034	(*Z*)-β-Ocimene	2.2	0.1	2.2	2.5	4.0	0.3	1.5	0.8	0.4	5.7	2.1	3.5
1044	1045	Phenylacetaldehyde	-	-	0.1	-	0.1	-	-	tr	-	0.1	0.1	tr
1045	1045	(*E*)-β-Ocimene	24.8	0.3	7.0	10.5	14.1	3.5	17.3	8.1	4.6	37.4	9.4	23.7
1051	1051	2,3,6-Trimethylhepta-1,5-diene	-	-	0.1	-	-	-	-	-	-	-	tr	tr
1057	1057	γ-Terpinene	0.4	0.1	0.1	0.1	13.1	-	-	0.1	0.2	3.8	0.2	-
1062	1073	*p*-Mentha-3,8-diene	-	-	0.2	0.1	-	0.2	0.1	0.2	0.4	-	-	-
1086	1086	Terpinolene	0.5	0.1	0.1	0.1	6.5	0.1	0.1	1.5	3.8	0.1	0.1	0.1
1090	1090	6,7-Epoxymyrcene	0.1	0.7	0.5	0.1	-	0.3	0.1	-	-	-	0.2	0.2
1091	1091	*p*-Cymenene	-	-	-	-	-	-	-	0.1	0.2	-	-	-
1091	1091	Rosefuran	0.1	-	0.4	0.2	-	0.1	0.1	0.1	0.1	-	0.2	0.4
1096	1097	α-Pinene oxide	0.1	-	0.5	0.3	-	0.2	0.1	0.1	tr	tr	0.2	0.5
1099	1098	Perillene	tr	0.5	0.3	0.1	-	0.2	tr	tr	-	-	-	-
1100	1101	Linalool	0.1	0.1	0.4	0.1	0.2	0.2	0.2	0.2	0.1	0.1	0.3	0.2
1104	1102	6-Methylhepta-3,5-dien-2-one	-	-	0.1	-	-	-	-	-	-	-	-	0.2
1121	1119	Myrcenol	0.2	-	-	-	-	-	-	-	-	-	-	-
1124	1124	*cis-p*-Menth-2-en-1-ol	-	-	-	-	-	-	-	-	-	0.1	0.1	-
1129	1128	(4*E*,6*Z*)-*allo*-Ocimene	-	-	-	0.2	0.2	-	0.3	0.1	0.1	0.2	0.1	0.2
1137	1138	Benzeneacetonitrile	-	-	-	-	-	-	-	-	-	0.1	-	-
1139	1139	(*E*)-Myroxide	0.2	-	0.3	0.2	0.2	0.1	0.1	tr	tr	-	0.4	0.6
1142	1142	*trans-p*-Menth-2-en-1-ol	-	-	-	-	-	-	-	-	-	-	0.1	-
1144	1142	Epoxyterpinolene	-	-	-	-	-	-	-	0.1	0.4	-	-	-
1148	1149	Camphor	0.1	-	-	-	-	-	-	-	-	-	-	-
1156	1156	Pentylbenzene	-	-	-	-	-	0.1	0.1	-	-	-	-	0.2
1156	1156	Camphene hydrate	0.3	0.2	0.1	0.1	-	-	-	-	-	-	-	-
1169	1169	Rosefuran epoxide	-	-	0.2	0.1	-	0.1	-	-	-	-	-	0.2
1173	1173	Borneol	0.2	tr	0.1	-	-	-	-	-	-	-	-	0.2
1179	1179	2-Isopropenyl-5-methyl-4-hexenal	-	-	-	-	0.1	-	-	0.1	0.2	-	-	-
1181	1180	Terpinen-4-ol	0.1	tr	-	-	-	-	-	-	tr	-	-	-
1186	1188	*p*-Methylacetophenone	-	0.1	0.1	-	-	0.1	-	tr	0.2	-	-	-
1187	1187	Cryptone	-	-	-	0.2	-	-	-	-	-	0.1	2.3	-
1188	1188	*p*-Cymen-8-ol	0.1	0.2	0.2	-	0.3	0.1	-	0.7	1.6	-	-	0.2
1195	1195	α-Terpineol	0.2	0.1	0.2	tr	-	0.1	0.1	0.1	0.1	0.1	0.2	0.2
1207	1206	Decanal	-	0.1	-	-	-	-	-	-	-	-	-	-
1208	1207	(3*E*)-Octenyl acetate	0.3	-	-	-	-	-	0.2	0.1	0.1	tr	0.1	0.1
1210	1211	Octyl acetate	-	0.3	-	-	-	-	-	-	-	-	-	-
1226	1231	(3Z)-Hexenyl 2-methylbutanoate	-	-	-	-	-	-	-	-	-	-	-	0.2
1229	1229	Thymyl methyl ether	-	0.1	-	-	1.2	-	-	-	0.1	-	-	-
1244	1244	Linalyl acetate	-	0.2	-	-	-	-	-	-	-	-	-	-
1254	1254	Piperitone	0.1	0.1	-	-	-	-	-	-	-	-	-	-
1272	1271	Decanol	-	2.7	-	-	-	-	-	-	-	-	-	-
1284	1282	Bornyl acetate	0.9	0.1	5.8	2.6	3.4	0.1	-	-	0.1	1.1	2.9	3.9
1286	---	Unidentified ^a^	-	-	0.8	tr	0.8	0.2	-	-	-	-	-	2.4
1304	1302	4-Methylhexyl 2-methylbutanoate	-	-	-	-	-	-	-	-	-	-	-	0.3
1307	1310	*cis*-3-Butyl-4-vinyl cyclopentene	0.1	-	-	-	-	-	-	-	-	-	-	-
1342	1343	2-(2,5-Dimethylphenyl)propanal	0.1	-	-	-	0.3	0.1	-	0.2	1.4	-	-	-
1346	1346	α-Terpinyl acetate	-	-	-	-	-	0.1	-	0.2	0.3	-	-	-
1350	1348	α-Longipinene	-	-	-	-	-	-	-	-	-	-	-	0.1
1369	1367	Cyclosativene	-	-	0.2	-	-	0.1	0.1	0.2	0.1	-	-	-
1370	1370	*iso*-Ledene	-	-	0.2	0.7	-	-	-	-	-	-	-	-
1374	1372	Longicyclene	-	-	0.3	0.1	-	0.1	0.5	0.2	0.2	-	-	0.2
1376	1375	α-Copaene	-	-	-	0.1	-	-	-	-	-	-	-	-
1389	1390	*trans*-β-Elemene	-	-	-	-	-	-	-	-	-	0.1	-	-
1405	1411	Thymohydroquinone dimethyl ether	-	-	-	-	-	-	-	-	-	-	-	0.1
1406	1406	α-Gurjunene	-	-	-	0.1	-	-	-	-	-	-	-	-
1408	1408	Decyl acetate	-	7.7	-	-	-	-	-	-	-	0.2	1.7	-
1408	1405	(*Z*)-β-Caryophyllene	-	-	-	-	-	-	-	-	-	-	-	0.6
1409	1411	Longifolene	-	-	3.9	1.3	1.5	1.7	4.9	2.7	2.8	-	0.7	2.7
1410	1415	β-Maaliene	-	-	-	0.1	-	-	-	-	-	-	-	-
1415	1414	α-Cedrene	-	-	0.2	0.1	-	-	0.1	0.1	0.1	-	-	-
1419	1417	(*E*)-β-Caryophyllene	0.1	0.1	0.5	0.4	0.7	0.2	0.2	0.3	0.2	0.5	0.4	0.1
1423	1423	β-Cedrene	-	-	0.1	0.1	-	-	0.1	0.1	0.1	tr	0.2	-
1427	1430	γ-Maaliene	-	-	-	0.2	-	-	-	-	-	-	-	-
1429	1430	γ-Elemene	-	-	-	-	-	-	-	-	-	0.1	-	-
1433	1432	*trans*-α-Bergamotene	0.1	-	0.3	0.3	0.1	0.1	0.2	0.2	0.1	0.1	0.5	0.1
1434	1435	α-Maaliene	-	-	-	0.1	-	-	-	-	-	-	-	-
1434	1436	α-Guaiene	-	-	-	-	-	-	-	0.1	0.1	0.3	-	-
1436	1433	β-Copaene	-	-	-	0.1	-	-	-	-	-	-	-	-
1438	1438	Aromadendrene	-	-	0.5	1.7	-	-	-	-	-	-	-	-
1439	1438	α-Guaiene	-	-	0.4	1.2	-	-	-	-	-	-	-	-
1440	1440	Guaia-6,9-diene	-	-	-	-	-	-	-	tr	tr	-	-	-
1446	1446	*cis*-Muurola-3,5-diene	-	-	-	0.3	-	-	-	-	-	-	-	-
1447	1447	Geranylacetone	-	-	-	-	-	-	-	-	-	0.1	0.1	0.1
1449	1449	α-Himachalene	-	-	0.3	0.1	-	0.1	0.4	0.3	0.2	-	0.2	0.2
1452	1452	(*E*)-β-Farnesene	-	-	0.5	0.4	0.2	0.4	0.3	0.8	0.5	0.1	0.8	0.2
1453	1453	Prezizaene	-	-	-	-	-	-	-	-	0.2	-	-	-
1455	1454	α-Humulene	0.8	0.9	tr	-	-	-	-	tr	0.1	0.1	0.1	-
1457	1451	Amorpha-4,11-diene	-	-	-	-	-	-	0.1	0.1	0.1	-	-	-
1459	1458	*allo*-Aromadendrene	-	-	0.2	0.5	-	-	-	-	-	-	-	-
1465	1465	Bornyl butyrate	-	-	-	-	-	-	-	-	0.1	-	-	-
1473	1474	Selina-4,11-diene	-	-	-	-	-	0.2	-	0.1	-	0.1	-	-
1472	1475	γ-Gurjunene	-	-	0.2	0.5	-	-	-	-	-	-	-	-
1474	1475	Dodecanol	-	0.2	-	-	-	-	-	-	-	-	-	-
1478	1479	α-Amorphene	-	0.1	-	-	-	-	-	0.9	0.5	-	-	-
1479	1480	γ-Himachalene	-	-	0.2	-	-	-	0.2	-	-	-	-	-
1481	1482	*ar*-Curcumene	-	-	0.1	-	-	0.1	0.5	0.3	0.7	-	-	-
1486	1488	δ-Selinene	-	-	-	0.3	-	-	-	-	-	-	-	-
1488	1489	β-Selinene	-	-	0.2	0.6	-	0.3	-	0.1	-	0.2	-	-
1489	1489	(*Z*,*E*)-α-Farnesene	3.2	1.4	-	-	-	0.5	0.2	-	-	-	-	-
1490	1491	Viridiflorene	-	-	1.0	5.9	0.1	-	-	-	-	-	-	-
1495	1497	α-Selinene	-	0.1	-	0.2	-	0.2	0.1	0.1	-	0.1	-	-
1498	1497	Capillene	-	-	-	-	0.5	-	-	-	-	-	-	-
1498	1500	α-Muurolene	-	0.1	-	-	-	-	0.1	tr	-	-	-	-
1499	1503	β-Himachalene	-	-	0.2	0.1	-	-	0.2	0.3	0.2	-	-	-
1501	1505	α-Bulnesene	-	-	0.2	0.5	-	-	-	-	-	0.2	-	-
1502	1504	Epizonarene	-	-	-	-	-	-	-	0.1	-	-	-	-
1503	1504	(*E*,*E*)-α-Farnesene	0.3	0.3	-	-	-	-	-	-	-	-	-	-
1504	1501	β-Dihydroagarofuran	-	-	-	-	-	0.2	-	0.1	-	-	-	-
1506	1508	β-Bisabolene	-	-	0.4	0.2	0.1	0.1	0.3	1.2	1.2	0.1	0.6	0.2
1509	1511	β-Curcumene	-	-	-	-	-	-	-	-	0.2	-	-	-
1509	1511	(*Z*)-γ-Bisabolene	-	0.2	-	0.1	-	1.9	3.7	1.0	0.1	-	-	-
1512	1514	Sesquicineole	-	-	-	-	-	-	-	0.1	0.1	-	-	-
1516	1518	Bornyl isovalerate	-	-	-	-	-	-	-	-	0.1	-	-	-
1517	1519	Nootkatene	-	-	-	0.3	-	-	-	-	-	-	-	-
1518	1518	δ-Cadinene	-	0.1	-	-	-	-	-	-	-	-	-	-
1523	1523	β-Sesquiphellandrene	-	-	-	-	-	-	-	-	-	-	0.2	-
1529	1528	(*E*)-γ-Bisabolene	-	0.1	-	-	-	-	0.2	0.4	0.1	-	-	-
1537	1540	Selina-4(15),7(11)-diene	-	-	-	-	-	-	-	0.6	0.2	0.4	-	-
1540	1540	(*E*)-α-Bisabolene	-	-	-	-	-	0.1	0.2	0.6	0.6	-	0.2	-
1542	1542	Selina-3,7(11)-diene	-	-	-	-	-	0.1	-	0.7	0.2	0.3	-	-
1548	1549	α-Elemol	-	-	-	-	-	-	-	-	-	-	0.6	-
1558	1560	Germacrene B	-	-	-	-	-	-	-	-	-	0.2	-	-
1560	1560	(*E*)-Nerolidol	0.2	1.3	0.9	0.4	0.3	2.8	0.9	0.6	0.3	0.1	1.0	0.8
1562	1564	*epi*-Globulol	-	-	0.5	1.3	-	-	-	-	-	-	-	-
1570	1568	Palustrol	-	-	-	0.9	-	-	-	-	-	-	-	-
1570	1570	Neryl 2-methylbutanoate	-	-	-	-	-	-	-	-	-	-	-	0.1
1576	1575	Caryolan-8-ol	-	-	0.3	-	-	-	-	0.1	-	-	-	-
1576	1578	Spathulenol	-	-	1.0	0.5	-	-	-	-	-	-	-	-
1581	1582	Caryophyllene oxide	-	-	0.4	-	-	0.1	-	-	-	-	-	-
1582	---	Unidentified ^b^	-	-	-	1.2	-	-	-	-	-	-	-	-
1585	1592	Globulol	-	-	2.3	5.6	0.1	0.1	-	-	-	-	-	-
1594	1594	Viridiflorol	-	-	-	0.3	-	-	-	-	-	-	-	-
1595	1596	(*E*)-β-Elemenone	0.2	-	-	-	-	-	-	-	-	-	-	-
1595	1593	Guaiol	-	-	-	-	-	0.1	-	0.1	-	0.3	-	-
1596	1596	Geranyl 2-methylbutanoate	-	-	-	-	-	-	-	-	-	-	-	0.4
1597	1596	Cubeban-11-ol	-	-	0.3	0.9	-	-	-	-	-	-	-	-
1602	1601	Longiborneol	-	-	-	0.2	-	-	-	-	-	-	-	-
1604	1604	Humulol	0.9	2.0	-	-	-	-	-	-	-	-	-	-
1605	1607	5-*epi*-7-*epi*-α-Eudesmol	-	-	-	-	-	0.1	-	-	-	-	-	-
1606	1609	Rosifoliol	-	-	-	0.4	-	-	-	-	-	-	-	-
1608	1610	Cedrol	-	-	0.2	0.2	-	-	-	-	-	-	0.4	-
1609	1613	Humulene epoxide II	-	0.4	-	-	-	-	-	-	-	-	-	-
1616	1613	Ledol	-	-	-	-	-	0.2	-	-	-	-	-	-
1624	1624	*epi*-γ-Eudesmol	-	-	1.0	2.6	0.1	2.6	1.6	1.4	-	-	-	-
1626	1624	Selina-6-en-4β-ol	-	0.1	-	-	-	-	-	-	-	-	-	-
1630	1632	γ-Eudesmol	-	-	-	-	-	4.1	2.6	1.9	tr	0.1	2.8	-
1637	1638	Gossonorol	-	-	-	-	-	0.1	0.1	-	-	-	-	-
1645	1645	Agarospirol (=Hinesol)	-	-	-	-	-	0.1	0.3	0.3	-	-	-	-
1648	1644	Selina-3,11-dien-6α-ol	-	-	-	-	-	-	-	-	-	0.1	-	-
1649	1649	3-Butylphthalide	-	0.1	-	-	-	-	-	-	-	-	-	-
1653	1650	Valerianol	-	-	-	-	-	1.1	0.9	1.1	-	-	-	-
1653	1655	α-Bisabolol oxide B	-	-	-	-	-	-	-	-	0.6	-	-	-
1654	1655	α-Cadinol	-	0.1	-	-	-	-	-	-	-	-	-	-
1655	1655	α-Eudesmol	-	-	-	-	-	6.5	4.4	3.6	-	0.6	5.0	0.2
1658	1660	*neo*-Intermedeol	-	-	-	-	-	0.3	-	0.2	-	-	-	-
1662	1664	*ar*-Turmerone	-	0.1	-	-	-	-	-	-	-	-	-	-
1670	1671	β-Bisabolol	-	-	-	-	-	0.1	1.5	-	0.1	-	-	-
1671	1668	Intermedeol	-	-	0.6	-	-	0.1	-	0.2	0.1	-	-	-
1675	1673	Bulnesol	-	-	-	0.5	-	-	-	-	-	-	-	-
1685	1686	*epi*-α-Bisabolol	-	-	0.3	-	-	0.2	0.3	-	-	-	0.3	-
1686	1688	α-Bisabolol	-	-	0.3	-	-	0.1	0.2	22.0	26.3	0.1	0.5	-
1692	1692	Civetone	-	0.1	-	-	-	0.1	-	-	-	-	-	-
1692	1694	Germacrone	-	-	-	-	-	-	-	-	-	-	0.3	-
1697	1696	Juniper camphor	-	-	-	-	-	-	-	0.2	-	0.2	-	-
1707	1701	β-Sinensal	-	-	-	-	-	-	0.2	-	-	-	-	-
1712	1711	14-Hydroxy-α-humulene	1.8	1.4	-	-	-	-	-	-	-	-	-	-
1712	1712	Senkyunolide (=Sedanenolide)	-	-	0.2	-	0.1	-	-	-	-	-	-	-
1720	1722	3-Isobutylidene phthalide	-	-	-	-	-	-	-	-	-	-	-	0.9
1722	1720	Longifolol	-	0.3	-	-	-	-	-	-	-	-	-	-
1741	1742	β-Bergamotol	1.5	0.4	-	-	-	-	-	-	-	-	-	-
1742	1742	(6*S*,7*R*)-Bisabolone	-	-	-	-	-	-	-	-	-	-	0.2	-
1760	1760	α-Sinensal	0.1	0.2	-	-	-	-	-	-	-	-	-	-
1767	1769	Benzyl benzoate	-	-	-	-	-	-	-	-	0.2	-	-	-
1767	1765	Eudesmyl acetate	-	-	-	-	-	2.3	0.2	0.7	-	-	-	-
1779	1776	δ-Cuparenol	-	-	-	-	-	-	-	-	-	4.8	-	-
1783	1784	Agarospyryl acetate	-	-	-	-	-	5.3	0.5	1.6	-	-	-	-
1878	1879	4-Phytadiene	-	0.1	-	-	-	-	-	-	-	-	-	-
1932	1933	Beyerene	-	0.1	0.1	-	-	0.1	-	-	-	-	-	-
1939	1938	Hexadecalact-16-one	-	-	-	-	-	-	-	-	-	-	1.8	-
1944	1946	*m*-Camphorene	-	0.3	0.2	0.1	-	0.3	-	-	-	-	-	-
1959	---	Unidentified ^c^	1.4	6.0	0.9	2.6	2.2	6.2	4.7	6.7	0.1	0.3	-	-
1959	1958	Palmitic acid	-	-	-	-	-	-	-	-	-	0.2	1.9	-
1961	---	2-Methyl-4,5-nonadiene ^d^	-	0.8	0.2	0.5	0.3	0.8	1.1	1.2	-	-	-	-
1979	1984	*p*-Camphorene	-	0.1	0.1	-	-	0.1	-	-	-	-	-	-
1981	1985	Vinyl palmitate	-	0.3	-	-	-	-	-	-	-	-	-	-
2028	---	Unidentified ^e^	-	0.7	0.2	0.5	1.0	0.4	0.3	0.7	-	-	-	-
2128	2128	Linoleic acid	-	-	-	-	-	0.2	-	-	-	-	0.2	-
2148	2143	Serratol	-	-	-	-	-	-	-	-	-	0.4	0.1	0.2
2164	2164	Ethyl linoleate	0.3	1.0	0.2	0.7	0.4	0.6	0.2	0.6	-	0.2	-	-
2203	---	Suberosin ^d^	-	-	0.9	0.4	2.2	-	-	-	-	-	-	-
2205	---	Unidentified ^f^	-	-	-	-	-	-	-	-	-	-	-	1.0
2300	2300	Tricosane	-	-	-	-	-	-	-	-	-	-	0.2	0.1
2500	2500	Pentacosane	0.1	0.1	0.2	-	0.2	tr	-	-	-	0.1	0.4	0.1
2700	2700	Heptacosane	-	-	0.2	-	0.4	-	-	-	-	0.2	0.4	0.1
		Monoterpene hydrocarbons	83.7	61.0	59.8	52.0	79.7	44.0	53.5	35.1	49.1	84.9	66.4	73.6
		Oxygenated monoterpenoids	3.0	2.5	8.9	3.8	5.4	1.5	0.8	1.4	3.1	1.5	7.0	7.4
		Sesquiterpene hydrocarbons	4.5	3.3	9.9	16.5	2.6	6.2	12.6	11.3	8.8	2.8	3.8	4.3
		Oxygenated sesquiterpenoids	4.7	6.3	8.2	13.8	0.5	26.5	13.8	34.1	27.5	6.2	11.0	1.0
		Diterpenes	0.0	0.6	0.4	0.1	0.0	0.4	0.0	0.0	0.0	0.4	0.1	0.2
		Benzenoid aromatics	0.1	0.3	2.0	1.2	3.6	0.3	3.6	0.2	1.8	0.2	0.3	1.5
		Others	1.2	13.8	3.9	3.3	3.1	9.7	8.6	7.5	8.0	3.0	10.6	4.3
		Total identified	97.2	87.9	93.0	90.6	94.8	88.5	92.9	89.6	98.3	98.9	99.3	92.3

RI_calc_ = retention index calculated with respect to a homologous series of *n*-alkanes on a ZB-5ms column. RI_db_ = reference retention index values obtained from the databases. Lm = *Lomatium multifidum*. OR = collected from eastern Oregon. ID = collected from western Idaho. tr = trace (<0.05%). - = not observed. ^a^ MS(EI): 96 (47%), 85 (53%), 81 (63%), 57 (100%), 55 (57%), 41 (29%). ^b^ MS(EI): 220 (92%), 205 (26%), 187 (21%), 177 (25%), 162 (33%), 159 (44%), 147 (77%), 135 (47%), 135 (48%), 133 (43%), 121 (42%), 119 (48%), 107 (70%), 105 (100%), 93 (60%), 91 (88%), 79 (58%), 77 (44%), 55 (40%), 43 (65%), 41 (80%). ^c^ MS(EI): 280 (1%), 237 (36%), 219 (4%), 149 (6%), 135 (15%), 121 (18%), 111 (13%), 97 (28%), 95 (25%), 83 (50%), 81 (33%), 69 (74%), 67 (30%), 57 (27%), 55 (100%), 43 (74%), 4 1(48%). ^d^ Reference RI not available, identification tentative. ^e^ MS(EI): 362 (3%), 313 (3%), 28 3(3%), 265 (4%), 251 (28%), 149 (7%), 135 (13%), 123 (14%), 121 (16%), 111 (22%), 109 (25%), 83 (68%), 81 (51%), 69 (81%), 67 (38%), 57 (47%), 55 (100%), 43 (98%), 41 (49%). ^f^ MS(EI): 244 (68%), 229 (100%), 214 (7%), 201 (7%), 189 (17%), 159 (12%), 131 (11%), 115 (10%), 77 (10%).

**Table 7:** Chemical compositions (%) of the essential oils of *Lomatium nudicaule* (Nutt.) J.M. Coult. & Rose.

RI_calc_	RI_db_	Compound	Ln#1	Ln#2	Ln#3	Ln#4	Ln#5	Ln#6	Ln#7
926	927	α-Thujene	tr	tr	0.1	tr	tr	tr	tr
933	933	α-Pinene	1.3	0.8	1.0	0.5	0.9	0.4	0.4
950	950	Camphene	tr	tr	tr	tr	tr	tr	tr
973	970	3,7,7-Trimethylcyclohepta-1,3,5-triene	-	0.1	0.2	-	-	-	-
974	972	Sabinene	0.4	0.1	0.1	0.2	0.2	0.1	0.1
979	978	β-Pinene	2.1	1.3	0.5	0.9	2.0	1.0	1.2
990	991	Myrcene	5.4	6.1	2.3	2.8	4.4	1.1	0.7
999	---	3,4-Dimethylenecyclopentanone ^a^	tr	0.1	-	-	-	-	-
1005	1004	*p*-Mentha-1(7),8-diene	0.4	0.3	0.3	0.2	0.3	0.2	0.1
1007	1007	α-Phellandrene	2.3	0.1	0.1	1.7	2.5	1.1	0.4
1010	1009	δ-3-Carene	0.8	5.2	12.6	1.3	1.9	0.5	0.2
1018	1018	α-Terpinene	0.1	tr	-	0.1	0.2	tr	tr
1020	1022	*m*-Cymene	0.2	tr	tr	-	-	-	-
1025	1025	*p*-Cymene	0.2	2.3	4.7	0.3	0.4	0.2	0.2
1030	1030	Limonene	0.1	1.0	2.5	0.5	0.4	0.2	0.3
1032	1031	β-Phellandrene	44.7	33.3	16.5	35.8	45.7	30.3	16.0
1035	1034	(*Z*)-β-Ocimene	0.2	0.8	1.7	0.2	0.1	0.3	1.8
1047	1046	(*E*)-β-Ocimene	9.6	3.6	6.0	3.3	3.7	5.9	9.9
1058	1057	γ-Terpinene	0.1	0.1	tr	0.1	0.1	tr	tr
1072	1072	*p*-Cresol	-	0.2	0.3	-	-	-	-
1074	1073	α-Pinene oxide	-	-	0.2	-	-	-	0.1
1082	1080	*m*-Cymenene	-	-	0.1	-	-	-	-
1086	1086	Terpinolene	1.4	1.2	1.7	1.8	2.9	0.7	0.3
1091	1091	*p*-Cymenene	-	0.8	1.4	-	tr	-	tr
1093	1091	Rosefuran	-	-	-	-	-	tr	0.1
1096	1097	α-Pinene oxide	-	-	-	tr	tr	tr	0.1
1100	1101	Linalool	0.6	0.2	0.2	0.2	0.2	0.5	-
1103	1101	6-Methylhepta-3,5-dien-2-one	-	-	0.1	-	-	-	-
1104	1104	2-Methylbutyl 2-methylbutyrate	-	-	-	tr	tr	0.1	-
1125	1124	*cis-p*-Menth-2-en-1-ol	0.1	0.1	0.1	0.1	0.1	tr	tr
1127	1131	Cyclooctanone	-	0.1	0.5	-	-	-	-
1128	1127	*allo*-Ocimene	-	-	-	-	-	-	0.1
1136	1130	(*Z*)-Myroxide	-	0.1	0.3	-	-	-	tr
1139	1339	3-Oxo-*p*-menth-1-en-7-al	tr	0.5	0.3	-	-	-	-
1140	1141	(*E*)-Myroxide	0.1	0.6	1.3	-	-	-	tr
1143	1142	*trans-p*-Menth-2-en-1-ol	0.1	-	-	0.1	0.1	tr	tr
1158	1156	Pentylbenzene	-	-	-	tr	tr	tr	tr
1159	1161	5-Pentylcyclohexa-1,3-diene	0.3	-	-	0.3	0.1	0.2	0.2
1175	1175	(3*E*,5*Z*)-1,3,5-Undecatriene	0.1	-	-	0.1	0.1	0.1	tr
1178	1180	(*E*)-Isocitral	-	-	-	-	tr	tr	-
1179	1180	Terpinen-4-ol	tr	0.1	-	tr	tr	tr	-
1183	1188	*p*-Methylacetophenone	-	0.2	0.6	-	-	-	-
1185	1187	Cryptone	0.8	6.4	7.7	0.4	0.5	0.4	0.3
1185	1185	(3*E*,5*E*)-1,3,5-Undecatriene	-	-	-	tr	tr	tr	tr
1194	1195	α-Terpineol	0.1	-	-	tr	tr	tr	-
1196	1195	*trans*-4-Caranone	0.1	0.7	0.3	-	-	-	-
1197	1195	*p*-Menth-3-en-7-al	-	0.5	0.3	0.1	0.1	0.1	tr
1201	1202	*cis*-Sabinol	0.1	0.4	0.6	0.1	0.1	0.1	tr
1208	1208	*trans*-Piperitol	tr	-	-	-	tr	-	-
1224	1227	Citronellol	0.6	0.5	1.0	0.3	0.4	0.1	-
1241	1247	Cuminal	-	0.3	0.2	-	-	-	-
1249	1254	*cis*-Piperitone epoxide	-	0.2	-	-	-	-	-
1263	1265	(2*E*)-Decenal	-	0.2	-	-	-	-	-
1272	1294	*p*-Mentha-1,5-diene-7-ol	0.1	0.5	0.4	0.1	0.1	0.1	-
1277	1277	Phellandral	-	0.3	0.2	-	-	-	-
1286	1286	α-Terpinen-7-al	tr	0.1	0.2	-	-	-	-
1291	1291	*p*-Cymen-7-ol	0.1	0.5	0.4	tr	tr	tr	-
1308	1313	Phthalic anhydride	-	1.2	1.7	-	-	-	-
1320	1318	3-Hydroxycineole	-	0.2	0.4	-	-	-	-
1321	1320	Methyl geranate	-	-	-	tr	tr	tr	-
1322	1318	4-Hydroxycryptone	-	0.1	0.2	-	-	-	-
1336	1336	δ-Elemene	-	-	-	-	tr	tr	tr
1339	1339	3-Oxo-*p*-menth-1-en-7-al	-	-	-	tr	tr	tr	tr
1343	---	Unidentified ^b^	0.4	2.0	2.2	0.6	0.5	0.8	0.9
1375	1375	α-Copaene	tr	-	-	-	-	-	-
1382	1383	*cis*-β-Elemene	-	-	-	-	tr	0.1	tr
1389	1390	*trans*-β-Elemene	0.1	-	-	0.5	0.3	0.7	0.8
1420	1417	(*E*)-β-Caryophyllene	0.1	0.1	0.3	0.3	0.3	0.8	0.1
1429	1427	γ-Elemene	0.6	0.1	0.2	3.0	1.7	3.9	3.7
1452	1452	(*E*)-β-Farnesene	0.1	0.7	0.7	0.1	0.2	0.2	0.4
1456	1454	α-Humulene	-	-	-	0.1	0.1	0.1	0.1
1465	1463	γ-Decalactone	-	-	-	0.1	tr	-	0.1
1479	1480	Germacrene D	0.1	-	-	0.7	0.4	1.0	0.9
1485	1483	Phenylethyl 2-methylbutyrate	0.1	0.1	-	0.1	0.1	0.1	0.1
1489	1489	β-Selinene	-	-	-	0.1	tr	0.1	0.1
1490	1493	Phenylethyl 3-methylbutanoate	-	-	-	-	-	-	0.1
1537	1540	Selina-4(15),7(11)-diene	-	-	-	-	-	0.1	-
1542	1541	(*E*)-α-Bisabolene	tr	0.1	tr	-	-	-	-
1549	1549	α-Elemol	-	-	-	-	-	-	0.1
1551	---	7-Hydroxypiperitone ^a^	-	0.3	0.3	-	-	-	-
1560	1557	Germacrene B	1.3	0.2	0.4	4.5	2.6	6.4	9.3
1574	1572	Citronellyl 2-methylbutyrate	0.1	0.4	0.3	-	0.1	0.1	0.1
1586	1587	Caryophyllene oxide	tr	0.1	0.4	-	tr	0.1	-
1596	1596	Geranyl 2-methylbutyrate	tr	0.1	-	-	tr	-	-
1633	2632	Tetracosanal	0.2	0.2	0.1	0.1	0.1	0.1	-
1654	1649	3-Butyl phthalide	-	0.1	0.1	-	-	-	-
1668	1669	(2*E*,6*Z*)-Farnesol	2.1	1.1	2.5	0.2	0.5	0.3	-
1670	1669	(3*Z*)-Butylidene phthalide	0.5	2.1	2.1	0.4	0.3	0.5	1.1
1675	1674	γ-Dodecalactone	-	-	-	0.1	tr	-	-
1684	1684	(2*Z*,6*Z*)-Farnesal	tr	0.1	0.2	-	-	-	-
1693	1692	(2*Z*,6*Z*)-Farnesol	0.1	-	-	-	-	-	-
1707	1705	14-Hydroxy-4,5-dihydrocaryophyllene	tr	-	-	-	-	-	-
1713	1716	(2*E*,6*E*)-Farnesol	-	0.1	0.1	-	-	-	-
1714	1712	(*Z*)-Sedanenolide	0.2	-	-	-	0.3	0.5	-
1719	1719	(3*E*)-Butylidene phthalide	0.2	0.6	1.4	0.3	-	-	0.4
1722	1722	3-Isobutylidene phthalide	-	-	2.8	0.2	-	-	-
1728	1730	(*Z*)-Ligustilide	17.4	8.6	5.6	33.2	22.4	33.0	47.1
1772	1772	α-Costol	-	-	0.3	0.2	0.1	0.4	-
1788	1790	(*E*)-Ligustilide	0.9	0.2	0.2	2.9	2.0	4.8	1.8
1807	---	Unidentified oxygenated sesquiterpenoid ^c^	0.5	2.2	1.3	0.6	0.3	0.8	0.9
1933	1928	Methyl linolenate	tr	0.1	-	-	-	-	-
1959	1958	Palmitic acid	0.2	0.4	0.3	-	-	-	-
2004	2005	Senkyunolide H	-	2.6	1.8	-	-	-	-
2036	2037	(*Z*)-Falcarinol	0.1	-	-	-	-	-	-
2128	2128	(*Z*,*Z*)-Linoleic acid	0.1	-	-	-	-	-	-
2300	2300	Tricosane	0.1	0.2	0.1	-	-	-	-
2439	2442	2-Methyltetracosane	-	-	1.2	-	-	-	-
2500	2500	Pentacosane	0.3	0.3	0.4	0.1	0.1	0.1	tr
2700	2700	Heptacosane	0.2	0.2	0.2	-	-	-	-
2800	2800	Octacosane	tr	-	-	-	-	-	-
2838	2833	Hexacosanal	0.7	-	-	-	-	-	-
		Monoterpene hydrocarbons	69.4	57.2	51.7	49.7	65.6	42.0	31.6
		Oxygenated monoterpenoids	2.7	13.1	14.8	1.2	1.6	1.4	0.6
		Sesquiterpene hydrocarbons	2.3	1.1	1.6	9.3	5.5	13.4	15.2
		Oxygenated sesquiterpenoids	2.2	1.3	3.5	0.4	0.6	0.8	0.1
		Benzenoid aromatics	19.2	13.4	14.6	37.0	24.8	38.4	1.7
		Others	2.5	4.3	4.7	0.7	0.6	1.1	49.2
		Total identified	98.3	90.4	90.8	98.3	98.7	97.2	98.3

RI_calc_ = retention index calculated with respect to a homologous series of *n*-alkanes on a ZB-5ms column. RI_db_ = reference retention index values obtained from the databases. Ln = *Lomatium nudicaule*. tr = trace (< 0.05%). - = not observed. ^a^ Reference RI not available, identification tentative. ^b^ MS(EI): 150 (48%), 106 (63%), 105 (44%), 78 (100%), 77 (45%), 52 (40%), 51 (42%), 49 (34%). ^c^ MS(EI): 222 (12%), 178 (9%), 166 (11%), 151 (17%), 137 (16%), 123 (17%), 110 (17%), 109 (17%), 95 (20%), 91 (16%), 83 (18%), 81 (24%), 67 (16%), 55 (100%), 53 (31%), 43 (14%), 41 (19%).

**Table 8:** Chemical compositions (%) of *Lomatium papilioniferum* J.A. Alexander & Whaley from northern Oregon and western Idaho.

RI_calc_	RI_db_	Compound	Lpap#1 (OR)	Lpap#2 (OR)	Lpap#3 (OR)	Lpap#4 (OR)	Lpap#5 (ID)	Lpap#6 (ID)	Lpap#7 (ID)	Lpap#8 (ID)
808	806	Hexanal	-	-	-	-	-	0.1	-	-
849	849	(2*E*)-Hexenal	0.2	tr	tr	tr	-	-	-	-
908	906	Heptanal	-	-	-	-	0.1	0.2	0.1	0.1
920	921	Hashishene	tr	-	-	-	-	-	-	-
922	923	Tricyclene	tr	tr	tr	tr	-	-	-	-
925	925	α-Thujene	tr	0.1	0.1	0.1	-	-	-	-
933	933	α-Pinene	0.7	1.8	2.1	2.1	tr	tr	0.1	0.2
947	948	α-Fenchene	tr	-	-	-	-	-	-	-
949	950	Camphene	1.7	0.8	1.0	0.7	0.2	tr	0.8	0.8
951	955	Propylbenzene	-	-	-	-	tr	0.5	0.1	0.7
965	963	2-Methyl-(3*E*)-octen-5-yne	0.2	-	-	-	-	-	0.2	0.2
970	972	Tetrahydrofurfuryl acetate	0.1	-	-	tr	-	-	-	-
972	971	Sabinene	0.2	0.7	0.9	0.6	-	-	-	-
978	978	β-Pinene	0.1	0.3	0.3	0.4	-	tr	tr	tr
984	984	6-Methylhept-5-en-2-one	-	-	-	-	tr	0.1	0.1	tr
989	989	Myrcene	27.5	5.4	6.8	3.1	0.2	0.3	0.4	0.6
990	990	Dehydro-1,8-cineole	-	tr	tr	tr	-	-	-	-
1000	1000	δ-2-Carene	tr	tr	tr	0.1	-	-	-	-
1005	1004	*p*-Mentha-1(7),8-diene	0.1	0.1	0.1	0.1	-	-	-	-
1007	1007	α-Phellandrene	-	1.1	1.1	-	tr	tr	tr	tr
1009	1009	δ-3-Carene	tr	0.4	0.4	0.1	tr	tr	tr	tr
1017	1017	α-Terpinene	-	0.3	0.3	-	0.1	0.2	0.3	0.3
1019	1016	Tetrahydro-2-furanmethanol acetate	-	-	-	0.1	-	-	-	-
1025	1025	*p*-Cymene	6.0	3.1	2.6	47.8	22.9	20.9	20.4	21.1
1029	1030	Limonene	2.4	1.6	2.0	3.0	0.2	0.2	0.6	0.6
1032	1031	β-Phellandrene	0.8	23.8	23.2	5.7	tr	tr	tr	tr
1032	1032	1,8-Cineole	-	-	-	-	tr	tr	tr	tr
1033	1033	Benzyl alcohol	-	-	-	-	-	tr	tr	tr
1035	1034	(*Z*)-β-Ocimene	0.2	0.2	0.2	0.2	tr	-	tr	tr
1043	1043	Phenylacetaldehyde	-	-	-	-	tr	tr	tr	tr
1046	1046	(*E*)-β-Ocimene	0.7	7.2	7.2	2.9	0.3	tr	0.1	tr
1051	1051	2,3,6-Trimethylhepta-1,5-diene	0.3	-	-	-	-	-	-	-
1058	1058	γ-Terpinene	0.1	30.9	28.6	3.1	7.3	9.1	10.3	15.1
1070	1069	*cis*-Linalool oxide (furanoid)	-	-	-	0.2	-	-	-	-
1071	1072	Dihydromyrcenol	0.2	-	-	-	-	-	-	-
1085	1086	Terpinolene	-	2.5	2.4	-	0.2	0.2	0.3	0.4
1086	1086	*trans*-Linalool oxide (furanoid)	-	-	-	0.1	-	-	-	-
1090	1090	6,7-Epoxymyrcene	0.7	-	-	0.4	-	-	-	-
1090	1091	*p*-Cymenene	-	-	-	-	tr	0.1	tr	tr
1091	1091	Rosefuran	0.4	-	-	0.1	-	-	-	-
1095	1097	α-Pinene oxide	0.1	-	-	0.1	-	-	-	-
1098	1098	Perillene	0.6	-	-	-	-	-	-	-
1100	1101	Linalool	0.5	1.4	1.8	0.4	tr	tr	0.1	tr
1103	1102	6-Methylhepta-3,5-dien-2-one	0.4	-	-	0.1	tr	tr	tr	tr
1105	1107	Nonanal	0.1	-	-	-	tr	tr	tr	tr
1120	1119	Myrcenol	0.2	-	-	-	-	-	-	-
1121	1121	Isopentylbenzene	-	-	-	-	-	tr	tr	tr
1122	1121	(3*E*,5*E*)-1,3,5-Undecatriene	-	-	-	-	-	tr	tr	tr
1124	1124	*cis-p*-Menth-2-en-1-ol	-	0.1	0.1	0.1	-	-	-	-
1127	1126	α-Campholenal	-	-	-	0.1	-	-	-	-
1129	1130	(*Z*)-Myroxide	0.1	-	-	tr	-	-	-	-
1133	1132	*cis*-Limonene oxide	-	-	-	tr	-	-	-	-
1137	1138	*trans*-Limonene oxide	-	-	-	0.1	-	-	-	-
1139	1139	(*E*)-Myroxide	0.5	-	-	0.4	-	-	-	-
1142	1142	Epoxyterpinolene	-	0.1	0.1	-	-	-	-	-
1143	1142	*trans-p*-Menth-2-en-1-ol	-	-	-	0.2	-	-	-	-
1147	1149	Camphor	0.2	-	-	0.1	-	-	-	-
1156	1156	Pentylbenzene	0.1	tr	tr	0.5	-	-	-	-
1156	1156	Camphene hydrate	0.2	-	-	-	-	-	tr	tr
1156	1156	Pentylbenzene	-	-	-	-	tr	0.1	-	tr
1157	1161	5-Pentylcyclohexa-1,3-diene	-	0.9	1.0	-	0.1	0.2	0.1	0.1
1159	1163	(2*E*)-Nonenal	-	-	-	-	-	tr	-	tr
1162	---	2-Propylphenyl methyl ether ^a^	-	-	-	-	-	0.3	-	0.1
1162	1162	(*E*,*E*)-2,6-Dimethyl-3,5,7-octatriene-2-ol	-	tr	tr	0.6	0.1	-	tr	-
1169	1169	Rosefuran epoxide	0.1	-	-	0.1	-	-	-	-
1172	1173	Borneol	0.1	-	-	-	-	-	-	-
1174	1175	(3*E*,5*Z*)-1,3,5-Undecatriene	-	tr	tr	-	-	-	-	-
1180	1180	Terpinen-4-ol	0.1	0.1	0.1	0.1	tr	-	0.1	0.1
1185	1188	*p*-Methylacetophenone	tr	-	-	-	-	-	-	-
1186	1189	*p*-Cymen-8-ol	-	-	-	-	0.1	0.6	0.1	tr
1187	1187	Cryptone	0.7	0.2	0.2	2.7	-	-	-	-
1189	1195	*trans*-4-Caranone	-	-	-	0.1	-	-	-	-
1195	1195	α-Terpineol	0.2	tr	tr	-	tr	-	tr	tr
1197	1205	*cis*-4-Caranone	-	tr	tr	0.3	-	-	-	-
1203	1202	*cis*-Sabinol	-	tr	tr	-	-	-	-	-
1207	1208	Verbenone	-	-	-	0.1	-	-	-	-
1207	1208	Decanal	0.1	-	-	-	-	-	-	-
1209	1209	*trans*-Piperitol	-	-	-	tr	-	-	-	-
1209	1207	(3*E*)-Octenyl acetate	0.2	-	-	-	-	-	-	-
1210	1211	Octyl acetate	0.1	-	-	-	-	-	-	-
1223	1231	*trans*-Chrysanthenyl acetate	-	tr	tr	-	-	-	-	-
1223	1224	Thymyl methyl ether	-	-	-	-	0.3	0.2	0.2	0.3
1238	1238	Carvacryl methyl ether	-	-	-	-	0.4	0.3	0.4	0.5
1242	1242	Cuminaldehyde	-	-	-	0.1	-	-	-	-
1254	1254	Piperitone	0.7	3.3	4.1	5.9	-	-	-	-
1265	1265	(2*E*)-Decenal	-	-	-	0.2	-	-	-	-
1273	1271	1-Decanol	2.2	-	-	-	-	-	-	-
1283	1282	Bornyl acetate	0.1	-	-	-	0.7	0.1	3.9	2.9
1285	1287	Limonene dioxide	-	-	-	0.1	-	-	-	-
1286	1287	*iso*-Bornyl acetate	-	-	-	-	-	-	tr	tr
1289	1289	Thymol	-	0.1	0.1	0.1	tr	tr	tr	tr
1290	1289	(9*Z*)-Tetradecenal	0.1	-	-	-	-	-	-	-
1292	1291	*p*-Cymen-7-ol	-	-	-	0.2	-	-	-	-
1298	1300	Carvacrol	-	tr	tr	0.1	tr	tr	tr	0.1
1314	1321	2-Methyl-5-(propan-2-ylidene)cyclohexane-1,4-diol	-	-	-	0.2	-	-	-	-
1323	1318	4-Hydroxycryptone	-	-	-	0.1	-	-	-	-
1339	1339	3-Oxo-*p*-Menth-1-en-7-al	-	-	-	0.1	-	-	-	-
1342	1343	2-(2,5-Dimethylphenyl)propanal	-	-	-	0.2	-	-	-	-
1350	1352	α-Longipinene	-	-	-	-	0.1	0.1	0.1	0.2
1369	1367	Cyclosativene	-	-	-	-	0.1	0.1	0.1	0.2
1372	1370	*iso*-Ledene	-	-	-	-	tr	0.1	tr	tr
1374	1372	Longicyclene	-	-	-	-	0.3	0.1	0.3	0.3
1375	1375	α-Copaene	-	-	-	-	-	0.1	0.1	0.1
1382	1383	2-*epi*-α-Funebrene	-	-	-	-	0.4	0.4	0.4	0.5
1384	1385	α-Duprezianene	-	-	-	-	0.9	0.7	0.8	0.8
1390	1390	β-Elemene	-	-	-	0.1	-	-	-	-
1392	1392	(*Z*)-Jasmone	-	tr	-	0.1	-	-	-	-
1399	1403	Methyl eugenol	-	-	-	-	0.7	4.0	0.1	0.1
1402	1403	α-Funebrene	-	-	-	-	0.3	0.3	0.3	0.3
1405	1403	di-*epi*-α-Cedrene	-	-	-	-	0.2	0.1	0.1	0.1
1408	1408	Isopropyl 4-ethylbenzoate	-	-	-	0.1	-	-	-	-
1408	1409	Decyl acetate	6.0	-	-	-	-	-	-	-
1409	1411	Longifolene	-	-	-	-	3.6	1.6	3.3	3.5
1416	1414	α-Cedrene	-	-	-	-	0.2	0.1	0.1	0.1
1417	1416	2-*epi*-β-Funebrene	-	-	-	-	0.3	0.3	0.4	0.4
1418	1417	(*E*)-β-Caryophyllene	0.1	tr	0.1	-	0.2	0.1	0.1	0.1
1424	1423	β-Cedrene	-	-	-	-	0.1	0.1	0.1	0.1
1426	1428	β-Duprezianene	-	-	-	-	0.1	0.1	0.1	0.1
1428	1427	γ-Elemene	-	tr	tr	-	-	-	-	-
1432	1432	*trans*-α-Bergamotene	0.1	0.2	0.2	-	-	-	-	-
1435	1433	*cis*-Thujopsene	-	-	-	-	0.1	0.1	0.1	0.1
1437	1437	*iso*-Bazzanene	-	-	-	-	0.2	0.1	0.1	0.1
1441	1442	Guaia-6,9-diene	-	-	-	-	0.1	-	-	-
1447	1447	Geranylacetone	-	-	-	-	0.3	0.7	0.4	0.4
1450	1449	α-Himachalene	1.2	-	-	-	0.6	0.2	0.5	0.4
1451	1451	(*E*)-β-Farnesene	0.1	tr	tr	-	1.5	1.1	1.7	2.1
1453	1450	1,2,2α,3,3,4,6,7,8,8α-Decahydro-2α,7,8-trimethylacenaphthylene	-	-	-	-	-	0.6	-	0.7
1455	1454	α-Humulene	1.4	tr	tr	-	-	-	-	-
1457	1457	*allo*-Aromadendrene	0.5	-	-	-	-	-	-	-
1457	1461	Amorpha-4,11-diene	-	-	-	-	1.1	0.6	0.7	0.6
1459	1456	7-Isopropenyl-1-methyl-4-methylenedecahydroazulene	-	-	-	-	0.1	0.1	0.1	0.1
1464	1463	γ-Decalactone	-	0.1	0.1	0.1	-	-	-	-
1466	1467	β-Acoradiene	-	-	-	-	0.1	tr	-	-
1473	1473	γ-Selinene	-	-	-	-	-	tr	-	0.1
1473	1474	10-*epi*-β-Acoradiene	-	-	-	-	0.1	-	-	-
1474	1476	1-Dodecanol	0.3	-	-	-	-	-	-	-
1474	1474	4-*epi*-α-Acoradiene	-	-	-	-	0.2	0.1	0.1	0.1
1476	1477	*trans*-Cadina-1(6),4-diene	-	-	-	-	0.2	0.1	-	0.1
1478	1479	α-Amorphene	0.2	-	-	-	0.6	tr	-	0.3
1478	1480	γ-Himachalene	-	-	-	-	-	-	0.2	-
1480	1480	Germacrene D	-	0.6	0.6	-	-	-	-	-
1480	1480	*ar*-Curcumene	-	-	-	-	0.8	0.6	0.7	0.6
1485	1488	4-*epi*-(*Z*)-Dihydroagarofuran	-	-	-	-	0.4	-	-	-
1488	1487	β-Selinene	-	-	-	-	-	0.1	0.1	0.1
1490	1491	δ-Decalactone	-	0.1	0.1	0.1	-	-	-	-
1490	1489	(*Z*,*E*)-α-Farnesene	-	-	-	-	0.2	0.2	0.1	0.4
1498	1499	Benzyl tiglate	-	tr	-	0.1	-	-	-	-
1498	1497	α-Muurolene	0.2	-	-	-	tr	0.1	0.1	0.1
1500	1503	β-Himachalene	-	-	-	-	0.3	0.1	0.3	0.3
1503	1504	α-Cuprenene	-	-	-	-	0.3	0.1	0.2	0.3
1506	1507	Geranyl isobutyrate	-	0.1	0.1	-	-	-	-	-
1506	1508	β-Bisabolene	0.1	-	-	-	-	-	-	-
1506	1506	α-Chamigrene	-	-	-	-	1.8	2.1	1.0	1.9
1508	1505	Cuparene	-	-	-	-	6.0	4.1	4.9	3.5
1512	1512	γ-Cadinene	0.3	tr	tr	-	-	-	-	-
1518	1523	β-Guaiene	0.7	-	-	-	-	-	-	-
1518	1518	δ-Cadinene	0.1	tr	tr	-	0.2	0.3	0.5	0.3
1519	1519	*trans*-Calamenene	-	-	-	-	0.1	0.1	tr	-
1522	1521	Zonarene	-	-	-	-	-	0.1	0.1	-
1526	1528	(*E*)-γ-Bisabolene	-	-	-	-	0.2	0.1	0.2	0.2
1528	1528	Kessane	-	tr	tr	0.1	-	0.1	tr	-
1534	1535	γ-Cuprenene	-	-	-	-	1.1	0.6	0.8	0.7
1541	1541	α-Calacorene	-	-	-	-	0.5	0.1	0.2	0.2
1546	1548	Elemicin	-	-	-	-	-	0.1	-	-
1547	1549	α-Agarofuran	-	-	-	-	-	0.1	0.1	0.1
1555	1555	(*Z*)-Dihydronerolidol	0.4	-	-	-	-	-	-	-
1558	1557	Germacrene B	-	tr	tr	-	-	-	-	-
1559	1560	(*E*)-Nerolidol	4.6	tr	tr	-	0.5	0.5	0.6	0.6
1569	1570	(*E*)-Dihydronerolidol	0.2	-	-	-	-	-	-	-
1575	1575	Caryolan-8-ol	0.2	-	-	-	0.1	tr	-	-
1576	1576	Spathulenol	-	tr	tr	-	-	-	-	-
1581	1587	Caryophyllene oxide	0.2	-	-	0.6	-	-	-	-
1582	1584	10-*epi*-Juneol	-	-	-	-	-	0.4	0.1	0.3
1594	1595	Geranyl 2-methylbutyrate	0.2	0.1	0.2	-	-	-	-	-
1596	1593	Guaiol	-	-	-	-	1.2	0.3	0.2	0.1
1598	1596	Humulene epoxide I	0.4	-	-	-	-	-	-	-
1602	1602	Geranyl isovalerate	-	0.1	0.1	-	-	-	-	-
1602	1601	Longiborneol	-	-	-	-	0.2	0.1	0.1	0.2
1603	1604	Humulol	8.4	tr	tr	-	-	-	-	-
1606	1606	Cedrol	-	-	-	-	0.6	0.7	0.5	0.5
1609	1611	Humulene epoxide II	3.8	-	-	0.3	-	-	-	-
1613	1612	5-*epi*-7-*epi*-β-Eudesmol	-	-	-	-	0.1	-	-	-
1621	1624	*epi*-γ-Eudesmol	0.2	-	-	-	-	-	-	-
1622	1624	10-*epi*-γ-Eudesmol	-	-	-	-	1.1	3.6	2.1	1.7
1625	1624	Selin-6-en-4β-ol	0.1	-	-	-	-	-	-	-
1625	1631	Eremoligenol	-	-	-	-	0.2	0.4	0.3	0.2
1627	1628	1-*epi*-Cubenol	-	-	-	-	-	0.1	0.1	-
1632	1633	γ-Eudesmol	-	-	-	-	0.2	0.4	0.2	0.2
1638	1644	*allo*-Aromadendrene epoxide	-	0.1	0.1	-	-	-	-	-
1641	1641	τ-Muurolol	0.1	-	-	-	-	-	-	-
1648	1649	3-Butylphthalide	2.2	0.8	0.8	5.1	-	-	-	-
1652	1646	Agarospirol (=Hinesol)	0.1	-	-	-	-	-	-	-
1653	1652	α-Eudesmol	0.2	-	-	-	-	-	-	-
1653	1657	Valerianol	-	-	-	-	0.7	2.5	1.2	1.1
1654	1655	α-Cadinol	0.3	-	-	-	-	-	-	-
1655	1656	β-Eudesmol	-	-	-	-	1.2	2.1	1.1	0.8
1661	1664	7-*epi*-α-Eudesmol	-	-	-	-	-	0.3	0.2	0.1
1665	1664	Bulnesol	-	-	-	-	0.8	0.1	-	-
1669	1671	β-Bisabolol	-	-	-	-	0.1	0.1	0.1	0.1
1672	1673	Cadalene	-	-	-	-	0.1	-	-	-
1676	1674	γ-Dodecalactone	-	-	-	0.1	-	-	-	-
1678	1676	1-Tetradecanol	-	-	-	-	-	0.2	-	0.1
1684	1686	*epi*-α-Bisabolol	0.2	-	-	-	-	-	-	-
1686	1686	α-Bisabolol	0.1	-	-	-	1.0	0.2	1.8	0.1
1691	1686	Octadec-(13*Z*)-enal	0.3	-	-	-	-	-	-	-
1712	1712	Sedanenolide (=Senkyunolide A)	1.5	10.8	10.7	1.8	-	-	-	-
1728	1730	(*Z*)-Ligustilide	-	tr	tr	-	-	-	-	-
1756	1756	Hexadec-(11*E*)-en-1-ol	0.4	-	-	-	-	-	-	-
1781	1776	2-Methyl-5-(1,2,2-trimethylcyclopentyl)phenol	-	-	-	-	30.5	31.5	29.3	24.9
1794	1796	Hexadec-(9*Z*)-enal	0.3	-	-	-	-	-	-	-
1802	---	Unidentified ^b^	-	-	-	-	0.8	0.7	1.8	1.3
1876	1878	Hexadec-(2*E*)-enal	0.5	-	-	-	-	-	-	-
1932	1933	Beyerene	0.3	tr	tr	-	-	-	-	-
1940	1938	Hexadecanolact-16-one	-	-	-	-	1.0	0.3	0.3	0.2
2035	2037	(*Z*)-Falcarinol	-	tr	-	-	-	-	-	-
2105	2106	Phytol	0.1	tr	tr	-	-	-	-	-
2300	2300	Tricosane	0.5	tr	tr	-	-	-	-	-
2500	2500	Pentacosane	0.5	0.1	tr	tr	-	-	-	-
		Monoterpene hydrocarbons	40.6	80.5	79.3	69.9	31.4	30.9	33.2	39.2
		Oxygenated monoterpenoids	5.4	5.4	6.7	10.3	1.5	1.2	4.7	3.8
		Sesquiterpene hydrocarbons	4.8	0.7	0.9	0.1	23.2	15.6	18.9	20.3
		Oxygenated sesquiterpenoids	19.7	0.1	0.1	0.9	39.1	43.4	37.6	30.9
		Diterpenoids	0.4	traces	traces	0.0	0.0	0.0	0.0	0.0
		Benzenoid aromatics	0.1	traces	traces	0.9	0.7	5.0	0.2	0.9
		Others	16.8	12.9	12.8	10.5	1.6	1.6	1.1	1.0
		Total identified	87.7	99.6	99.7	92.4	97.3	97.6	95.7	96.1

RI_calc_ = retention index calculated with respect to a homologous series of *n*-alkanes on a ZB-5ms column. RI_db_ = reference retention index values obtained from the databases. Lpap = *Lomatium papilioniferum*. OR = collected from northern Oregon. ID = collected from western Idaho. tr = trace (< 0.05%). - = not observed. ^a^ Reference RI not available, identification tentative. ^b^ MS(EI): 220 (2%), 205 (3%), 136 (100%), 121 (90%), 107 (17%), 105 (15%), 93 (42%), 91 (23%), 79 (18%), 77 (10%), 67 (8%), 55 (10%), 41 (14%).

**Table 9:** Comparison of *Lomatium* essential oil components by analysis of variance (ANOVA) followed by Tukey’s post hoc test.

*Lomatium* Species	Component Percentage (Means ± Standard Deviations)							
	Limonene	Sabinene	α-Pinene	β-Phellandrene	Myrcene	β-Pinene	Cryptone	(*E*)-β-Ocimene
*Lomatium anomalum*	1.2 ± 0.5 ^b^	48.7 ± 1.0 ^a^	27.7 ± 8.6 ^a^	1.6 ± 0.6 ^c^	0.9 ± 0.6 ^b^	3.0 ± 0.8 ^b^	0.0 ^b^	0.5 ± 0.3 ^ab^
*Lomatium dissectum* var. *dissectum*	traces ^b^	traces ^c^	traces ^b^	traces ^c^	traces ^b^	0.1 ± 0.1 ^b^	0.0 ^b^	traces ^b^
*Lomatium multifidum*	4.2 ± 3.8 ^b^	0.1 ± 0.1 ^c^	0.4 ± 0.4 ^b^	4.0 ± 7.8 ^c^	30.7 ± 13.2 ^a^	0.1 ± 0.1 ^b^	0.2 ± 0.6	13.4 ± 10.8 ^a^
*Lomatium nudicaule*	0.7 ± 0.9 ^b^	0.2 ± 0.1 ^c^	0.8 ± 0.3 ^b^	31.8 ± 12.0 ^a^	3.3 ± 2.1 ^b^	1.3 ± 0.6 ^b^	2.4 ± 3.3 ^ab^	6.0 ± 2.8 ^ab^
*Lomatium packardiae*	60.9 ± 10.1 ^a^	0.7 ±1.0 ^c^	1.0 ± 0.6 ^b^	5.3 ± 0.8 ^bc^	3.1 ± 0.5 ^b^	1.6 ± 0.8 ^b^	0.1 ± 0.1 ^b^	1.0 ± 1.1 ^b^
*Lomatium papilioniferum* (Idaho)	0.4 ± 0.2 ^b^	0.0 ^c^	0.1 ± 0.1 ^b^	traces ^c^	0.4 ± 0.2 ^b^	traces ^b^	0.0 ^b^	0.1 ± 0.1 ^b^
*Lomatium papilioniferum* (Oregon)	2.2 ± 0.6 ^b^	0.6 ± 0.3 ^c^	1.7 ± 0.7 ^b^	13.4 ± 11.9 ^abc^	10.7 ± 11.3 ^b^	0.3 ± 0.2 ^b^	0.9 ± 1.2 ^b^	4.5 ± 3.3 ^ab^
*Lomatium triternatum* var. *triternatum*	2.5 ± 2.0 ^b^	5.9 ± 3.9 ^b^	5.1 ± 4.3 ^b^	26.5 ± 23.5 ^ab^	9.9 ± 6.1 ^b^	7.7 ± 6.3 ^a^	7.5 ± 9.2 ^a^	5.8 ± 5.1 ^ab^
	**octyl acetate**	**decyl acetate**	***p*-cymene**	**γ-terpinene**	**sedanenolide**	**MTMCP**	**(*Z*)-ligustilide**	**δ-3-carene**
*Lomatium anomalum*	0.0 ^b^	0.0 ^b^	0.5 ± 0.3 ^b^	4.8 ± 2.2 ^ab^	0.0 ^b^	traces ^b^	0.4 ± 0.4 ^b^	traces ^b^
*Lomatium dissectum* var. *dissectum*	42.6 ± 3.9 ^a^	40.4 ± 4.8 ^a^	traces ^b^	traces ^b^	0.0 ^b^	0.0 ^b^	traces ^b^	0.0 ^b^
*Lomatium multifidum*	traces ^b^	0.8 ± 2.2 ^b^	2.0 ± 4.1 ^b^	1.5 ± 3.8 ^b^	traces ^b^	0.0 ^b^	0.0 ^b^	0.0 ^b^
*Lomatium nudicaule*	0.0 ^b^	0.0 ^b^	1.2 ± 1.7 ^b^	0.1 ± 0.1 ^b^	0.1 ± 0.1 ^b^	0.0 ^b^	23.9 ± 14.8 ^a^	3.2 ± 4.5 ^a^
*Lomatium packardiae*	0.0 ^b^	0.0 ^b^	0.1 ± 0.0 ^b^	0.1 ± 0.1 ^b^	traces ^b^	0.1 ± 0.1 ^b^	16.2 ± 3.0 ^a^	traces ^b^
*Lomatium papilioniferum* (Idaho)	0.0 ^b^	0.0 ^b^	21.4 ± 1.1 ^a^	10.5 ± 3.4 ^ab^	0.0 ^b^	29.0 ± 2.9 ^a^	0.0 ^b^	traces ^b^
*Lomatium papilioniferum* (Oregon)	traces ^b^	1.5 ± 3.0 ^b^	14.9 ± 22.0 ^ab^	15.7 ± 16.4 ^a^	6.2 ± 5.3 ^a^	0.0 ^b^	traces ^b^	0.2 ± 0.2 ^b^
*Lomatium triternatum* var. *triternatum*	0.0 ^b^	0.0 ^b^	2.4 ± 1.8 ^b^	0.2 ± 0.2 ^b^	0.2 ± 0.2 ^b^	0.0 ^b^	0.0 ^b^	traces ^b^

For each column, means that do not share a letter are significantly different (*p* < 0.05). MTMCP = 2-methyl-5-(1,2,2-trimethylcyclopentyl)phenol.

**Table 10:** Enantiomeric distribution of chiral terpenoid components of *Lomatium* species.

***Lomatium* Species**	**Enantiomeric Distribution, (+): (−)**									
	**α-Thujene**	**α-Pinene**	**Camphene**	**Sabinene**	**β-Pinene**	**α-Phellandrene**	**Limonene**	**β-Phellandrene**	***cis*-Sabinene Hydrate**	
*Lomatium triternatum* complex										
La#1	0.0: 100.0	98.8: 1.2	-	95.8: 4.2	68.0: 32.0	-	60.2: 39.8	94.9: 5.1	92.7: 7.3	
La#2	0.0: 100.0	98.2: 1.8	-	95.1: 4.9	0.0: 100.0	-	56.3: 43.7	93.8: 6.2	92.9: 7.1	
La#3	0.0: 100.0	98.5: 1.5	-	95.8: 4.2	0.0: 100.0	-	52.5: 47.5	95.0: 5.0	92.1: 7.9	
Lpack#1	-	47.2: 52.8	-	-	66.7: 33.3	100.0: 0.0	99.3: 0.7	84.9: 15.1	-	
Lpack#2	-	29.5: 70.5	-	-	28.8: 71.2	100.0: 0.0	99.1: 0.9	99.8: 0.2	-	
Lpack#3	-	6.3: 93.7	-	5.1: 94.9	2.8: 97.2	94.3: 5.7	98.9: 1.1	96.0: 4.0	-	
Lpack#4	-	6.6: 93.4	-	14.1: 85.9	2.1: 97.9	98.1: 1.9	99.1: 0.9	99.2: 0.8	-	
Ltt#1	-	51.2: 48.8	-	5.7: 94.3	27.5: 72.5	100.0: 0.0	29.8: 70.2	96.4: 3.6	-	
Ltt#2	-	5.4: 94.6	-	8.2: 91.8	2.7: 97.3	-	20.2: 79.8	86.1: 13.9	-	
Ltt#3	0.0: 100.0	3.3: 96.7	8.8: 91.2	3.8: 96.2	2.2: 97.8	100.0: 0.0	24.7: 75.3	97.0: 3.0	-	
*Lomatium grayi* complex										
Lpap#1 (OR)	-	30.3: 69.7	20.0: 80.0	-	-	-	26.8: 73.2	77.0: 23.0	-	
Lpap#2 (OR)	64.8: 35.2	24.0: 76.0	15.9: 84.1	53.1: 46.9	14.5: 85.5	87.1: 12.9	13.9: 86.1	85.6: 14.4	-	
Lpap#3 (OR)	62.2: 37.8	24.3: 75.7	15.5: 84.5	53.8: 46.2	16.0: 84.0	81.6: 18.4	12.0: 88.0	80.9: 19.1	-	
Lpap#4 (OR)	61.1: 38.9	13.7: 86.3	13.8: 86.2	70.6: 29.4	8.8: 91.2	-	11.3: 88.7	78.8: 21.2	-	
Lpap#5 (ID)	-	-	25.9: 74.1	-	-	-	34.5: 65.5	-	-	
Lpap#6 (ID)	-	52.5: 47.5	-	-	-	-	54.4: 45.7	-	-	
Lpap#7 (ID)	-	45.2: 54.8	27.4: 72.6	-	-	-	37.2: 62.8	-	-	
Lpap#8 (ID)	-	43.1: 56.9	23.4: 76.6	-	-	-	31.5: 68.5	-	-	
*Lomatium dissectum* complex										
Ld#1	-	60.2: 39.8	-	-	39.8: 60.2	-	79.3: 20.7	100.0: 0.0	-	
Ld#2	-	33.1: 66.9	-	-	63.5: 36.5	-	62.0: 38.0	100.0: 0.0	-	
Ld#3	-	54.9: 45.1	-	-	-	-	100.0: 0.0	100.0: 0.0	-	
Ld#4	-	87.8: 12.2	-	-	95.7: 4.3	-	59.1: 40.9	100.0: 0.0	-	
Ld#5	-	36.5: 63.5	-	-	79.8: 20.2	-	56.9: 43.1	100.0: 0.0	-	
Lm#1 (OR)	-	33.4: 66.6	19.8: 80.2	-	-	-	24.7: 75.3	-	-	
Lm#2 (OR)	-	35.9: 64.1	21.7: 78.3	-	-	-	35.6: 64.4	-	-	
Lm#3 (OR)	-	36.6: 63.4	26.9: 73.1	-	-	-	34.9: 65.1	-	-	
Lm#4 (OR)	-	42.4: 57.6	28.1: 71.9	-	-	-	35.6: 64.4	100.0: 0.0	-	
Lm#5 (OR)	-	46.2: 53.8	31.3: 68.7	-	-	-	46.5: 53.5	100.0: 0.0	-	
Lm#6 (OR)	-	45.1: 54.9	76.4: 23.6	-	-	-	55.5: 44.5	-	-	
Lm#7 (ID)	-	-	-	-	-	-	38.7: 61.3	-	-	
Lm#8 (ID)	-	41.9: 48.1	80.9: 19.1	-	-	-	51.7: 48.3	-	-	
Lm#9 (ID)	-	40.5: 59.5	54.4: 45.6	-	-	-	47.6: 52.4	-	-	
Lm#10 (OR)	-	65.5: 34.5	30.5: 69.5	-	-	100.0: 0.0	43.6: 56.4	100.0: 0.0	-	
Lm#11 (OR)	-	52.2: 47.8	24.5: 75.5	-	-	-	34.9: 65.1	100.0: 0.0	-	
Lm#12 (OR)	-	21.1: 78.9	24.7: 75.3	-	-	-	42.8: 57.2	-	-	
*Lomatium nudicaule*										
Ln#1	-	92.9: 7.1	-	100.0: 0.0	100.0: 0.0	100.0: 0.0	41.1: 58.9	100.0: 0.0	-	
Ln#2	-	89.9: 10.1	-	-	94.1: 5.9	-	43.6: 56.4	99.9: 0.1	-	
Ln#3	-	93.7: 6.3	-	-	90.3: 9.7	-	48.8: 51.2	100.0: 0.0	-	
Ln#4	-	87.8: 12.2	-	-	92.3: 7.7	100.0: 0.0	43.7: 56.3	99.9: 0.1	-	
Ln#5	-	91.5: 8.5	-	-	96.7: 3.3	100.0: 0.0	44.8: 55.2	99.9: 0.1	-	
Ln#6	-	88.3: 11.7	-	-	94.6: 5.4	100.0: 0.0	43.2: 56.8	99.9: 0.1	-	
Ln#7	-	90.9: 9.1	-	-	92.0: 8.0	100.0: 0.0	49.0: 51.0	100.0: 0.0	-	
***Lomatium* Species**	**Enantiomeric Distribution, (+): (−)**									
	**Linalool**	***trans*** **-Sabinene Hydrate**	**Terpinen-4-ol**	**α-Terpineol**	**Piperitone**	**(*E*)-β-Caryophyllene**	**Germacrene D**	**β-Bisabolene**	**δ-Cadinene**	**(*E*)-Nerolidol**
*Lomatium triternatum* complex										
La#1	-	93.1: 6.9	72.0: 28.0	59.3: 40.7	-	0.0: 100.0	0.0: 100.0	-	-	-
La#2	-	95.4: 4.6	71.8: 28.2	55.8: 44.2	-	0.0: 100.0	0.0: 100.0	-	-	-
La#3	-	95.7: 4.3	71.8: 28.2	55.5: 44.5	-	0.0: 100.0	0.0: 100.0	-	-	-
Lpack#1	-	-	-	-	-	-	-	-	-	-
Lpack#2	-	-	-	-	-	-	80.6: 19.4	-	-	-
Lpack#3	-	-	29.4: 70.6	-	-	0.0: 100.0	24.6: 75.4	-	-	-
Lpack#4	-	-	-	-	-	-	30.9: 69.1	-	-	-
Ltt#1	56.8: 43.2	-	35.7: 64.3	-	-	-	0.0: 100.0	-	-	-
Ltt#2	67.5: 32.5	-	-	-	-	-	-	-	-	-
Ltt#3	-	-	30.4: 69.6	-	-	0.0: 100.0	0.0: 100.0	-	-	-
*Lomatium grayi* complex										
Lpap#1 (OR)	67.7: 32.3	-	-	29.3: 70.7	-	-	-	-	-	7.4: 92.6
Lpap#2 (OR)	88.6: 11.4	-	67.6: 32.4	26.2: 73.8	0.3: 99.7	-	0.0: 100.0	-	-	-
Lpap#3 (OR)	90.1: 9.9	-	69.5: 30.5	27.2: 72.8	0.3: 99.7	-	0.0: 100.0	-	-	-
Lpap#4 (OR)	78.7: 21.3	-	67.5: 32.5	-	0.4: 99.6	-	-	-	-	-
Lpap#5 (ID)	-	-	-	-	-	-	-	-	-	30.8: 69.2
Lpap#6 (ID)	-	-	-	-	-	0.0: 100.0	-	-	100.0: 0.0	36.1: 63.9
Lpap#7 (ID)	-	-	-	-	-	0.0: 100.0	-	-	100.0: 0.0	28.8: 71.2
Lpap#8 (ID)	-	-	-	-	-	0.0: 100.0	-	-	100.0: 0.0	26.9: 73.1
*Lomatium dissectum* complex										
Ld#1	-	-	-	-	-	0.0: 100.0	-	-	-	-
Ld#2	-	-	-	-	-	0.0: 100.0	-	-	-	-
Ld#3	-	-	-	-	-	0.0: 100.0	-	-	-	-
Ld#4	-	-	-	-	-	0.0: 100.0	-	-	-	-
Ld#5	-	-	-	-	-	0.0: 100.0	-	-	-	-
Lm#1 (OR)	-	-	-	29.6: 70.4	-	0.0: 100.0	-	-	-	-
Lm#2 (OR)	-	-	-	30.3: 69.7	-	0.0: 100.0	-	-	-	7.7: 92.3
Lm#3 (OR)	-	-	-	26.4: 73.6	-	0.0: 100.0	-	-	-	5.0: 95.0
Lm#4 (OR)	-	-	-	-	-	0.0: 100.0	-	-	-	-
Lm#5 (OR)	-	-	-	-	-	0.0: 100.0	-	-	-	6.5: 93.5
Lm#6 (OR)	-	-	-	-	-	0.0: 100.0	-	-	-	15.2: 84.8
Lm#7 (ID)	-	-	-	-	-	0.0: 100.0	-	100.0: 0.0	-	18.7: 81.3
Lm#8 (ID)	-	-	-	28.2: 71.8	-	0.0: 100.0	-	25.6: 74.4	-	14.7: 85.3
Lm#9 (ID)	-	-	-	30.3: 69.7	-	0.0: 100.0	-	17.1: 82.9	-	11.0: 89.0
Lm#10 (OR)	-	-	-	-	-	0.0: 100.0	-	-	-	-
Lm#11 (OR)	-	-	-	-	-	0.0: 100.0	-	100.0: 0.0	-	8.3: 91.7
Lm#12 (OR)	-	-	-	-	-	0.0: 100.0	-	100.0: 0.0	-	8.5: 91.5
*Lomatium nudicaule*										
Ln#1	27.1: 72.9	-	-	-	-	0.0: 100.0	100.0: 0.0	-	-	-
Ln#2	37.1: 62.9	-	-	-	-	-	-	-	-	-
Ln#3	32.0: 68.0	-	-	-	-	-	-	-	-	-
Ln#4	19.6: 80.4	-	-	-	-	0.0: 100.0	93.4: 6.6	-	-	-
Ln#5	22.6: 77.4	-	-	-	-	0.0: 100.0	94.3: 5.7	-	-	-
Ln#6	10.7: 89.3	-	-	-	-	0.0: 100.0	94.9: 5.1	-	-	-
Ln#7	-	-	-	-	-	0.0: 100.0	-	-	-	-

La = *Lomatium anomalum*, Lpack = *Lomatium packardiae*, Ltt = *Lomatium triternatum* var. *triternatum*, Lpap = *Lomatium papilioniferum*, OR = sample collected in Oregon, ID = sample collected in Idaho, Ld = *Lomatium dissectum*, Lm = *Lomatium multifidum*, Ln = *Lomatium nudicaule*, - = not observed.

**Table 11:** Enantiomer percentages and significance for chiral monoterpenoids in *Lomatium* species based on ANOVA/Tukey.

	Enantiomer Percentage (Means ± Standard Deviations)					
*Lomatium* Species	(+)-α-Pinene	(−)-Camphene	(+)-Sabinene	(+)-β-Pinene	(+)-Limonene	(+)-Linalool
*Lomatium anomalum*	98.5 ± 0.3 ^a^	-	95.6 ± 0.4 ^a^	22.7 ± 39.3 ^bc^	56.3 ± 3.9 ^bc^	-
*Lomatium packardiae*	22.4 ± 19.8 ^c^	-	9.6 ± 6.4 ^c^	25.1 ± 30.4 ^bc^	99.1 ± 0.2 ^a^	-
*Lomatium triternatum* var. *triternatum*	20.0 ± 27.1 ^c^	-	5.9 ± 2.2 ^c^	10.8 ± 14.5 ^c^	24.9 ± 4.8 ^de^	62.2 ± 7.6 ^a^
*Lomatium papilioniferum* (Oregon)	23.1 ± 6.9 ^c^	83.7 ± 2.6 ^a^	59.2 ± 9.9 ^b^	13.1 ± 3.8 ^c^	16.0 ± 7.3 ^e^	81.3 ± 10.4 ^a^
*Lomatium papilioniferum* (Idaho)	46.9 ± 4.9 ^bc^	74.4 ± 2.0 ^a^	-	-	39.4 ± 10.3 ^cd^	-
*Lomatium dissectum*	54.5 ± 21.9 ^b^	-	-	69.7 ± 23.9 ^ab^	71.5 ± 18.2 ^b^	-
*Lomatium multifidum*	41.9 ± 11.2 ^bc^	61.9 ± 22.1 ^a^	-	-	41.0 ± 8.6 ^cd^	-
*Lomatium nudicaule*	90.7 ± 2.2 ^a^	-	-	94.3 ± 3.3 ^a^	44.9 ± 3.0 ^cd^	24.9 ± 9.4 ^b^

For each column, means that do not share a letter are significantly different (*p* < 0.05). - = not observed.

**Table 12:** Collection and hydrodistillation details for *Lomatium* species.

*Lomatium* Species (Voucher Number)	Sample #	Collection Site	Collection Date	Mass Aerial Parts (g)	Mass Essential Oil (g)	Essential Oil Color	%
*Lomatium anomalum* Jones ex J.M. Coult. & Rose (voucher WNS-La-5379)	#1	Near Grangeville, Idaho (45°55′29″ N, 116°8′19″ W, 1042 m asl)	2 June 2022	72.25	1.214	pale yellow	1.68
*Lomatium anomalum* Jones ex J.M. Coult. & Rose	#2	Near Grangeville, Idaho (45°52′34″ N, 116°13′40″ W, 1079 m asl)	30 May 2024	108.12	1.692	colorless	1.57
*Lomatium anomalum* Jones ex J.M. Coult. & Rose	#3	Near Grangeville, Idaho (45°52′34″ N, 116°13′40″ W, 1079 m asl)	30 May 2024	97.72	1.594	colorless	1.63
*Lomatium dissectum* (Nutt.) Mathias & Constance (voucher WNS-Ld-0181)	#1	Near Grangeville, Idaho (45°52′34″ N, 116°13′40″ W, 1079 m asl)	30 May 2024	94.80	2.401	colorless	2.53
*Lomatium dissectum* (Nutt.) Mathias & Constance	#2	Near Grangeville, Idaho (45°50′24″ N, 116°14′6″ W, 1275 m asl)	30 May 2024	113.31	3.107	colorless	2.74
*Lomatium dissectum* (Nutt.) Mathias & Constance	#3	Near Grangeville, Idaho (45°50′24″ N, 116°14′6″ W, 1275 m asl)	30 May 2024	238.04	4.623	colorless	1.94
*Lomatium dissectum* (Nutt.) Mathias & Constance	#4	Near Grangeville, Idaho (45°50′24″ N, 116°14′6″ W, 1275 m asl)	30 May 2024	129.21	3.370	colorless	2.61
*Lomatium dissectum* (Nutt.) Mathias & Constance	#5	Near Grangeville, Idaho (45°50′24″ N, 116°14′6″ W, 1275 m asl)	30 May 2024	198.53	4.070	colorless	2.05
*Lomatium multifidum* (Nutt.) R.P. McNeill & Darrach (voucher WNS-Lm-7137)	#1	Between Boggs Junction and Arlington, Oregon (45°41′23″ N, 120°30′0″ W, 97 m asl)	17 April 2023	176.41	4.712	yellow	2.67
*Lomatium multifidum* (Nutt.) R.P. McNeill & Darrach	#2	Between Boggs Junction and Arlington, Oregon (45°41′23″ N, 120°30′0″ W, 97 m asl)	17 April 2023	89.14	1.699	yellow	1.91
*Lomatium multifidum* (Nutt.) R.P. McNeill & Darrach	#3	Leslie Gulch, Oregon (43°18′22″ N, 117°17′31″ W, 955 m asl)	27 May 2023	64.18	3.429	yellow	5.34
*Lomatium multifidum* (Nutt.) R.P. McNeill & Darrach	#4	Leslie Gulch, Oregon (43°18′22″ N, 117°17′31″ W, 955 m asl)	27 May 2023	83.37	4.492	yellow	5.39
*Lomatium multifidum* (Nutt.) R.P. McNeill & Darrach	#5	Leslie Gulch, Oregon (43°18′22″ N, 117°17′31″ W, 955 m asl)	27 May 2023	62.89	1.288	yellow	2.05
*Lomatium multifidum* (Nutt.) R.P. McNeill & Darrach	#6	Near Prairie, Idaho (43°32′33″ N, 115°48′14″ W, 1143 m asl)	25 May 2023	38.87	2.141	yellow	5.51
*Lomatium multifidum* (Nutt.) R.P. McNeill & Darrach	#7	Near Prairie, Idaho (43°32′33″ N, 115°48′14″ W, 1143 m asl)	25 May 2023	79.81	4.907	yellow	6.148
*Lomatium multifidum* (Nutt.) R.P. McNeill & Darrach	#8	Near Prairie, Idaho (43°32′33″ N, 115°48′14″ W, 1143 m asl)	25 May 2023	43.88	1.583	yellow	3.61
*Lomatium multifidum* (Nutt.) R.P. McNeill & Darrach	#9	Near Prairie, Idaho (43°32′33″ N, 115°48′14″ W, 1143 m asl)	25 May 2023	58.09	2.537	yellow	4.37
*Lomatium multifidum* (Nutt.) R.P. McNeill & Darrach	#10	Lake Owyhee, Oregon (43°36′33″ N, 117°15′15″ W, 841 m asl)	8 May 2024	97.41	2.578	colorless	2.65
*Lomatium multifidum* (Nutt.) R.P. McNeill & Darrach	#11	Lake Owyhee, Oregon (43°36′33″ N, 117°15′15″ W, 841 m asl)	8 May 2024	69.77	1.116	pale yellow	1.60
*Lomatium multifidum* (Nutt.) R.P. McNeill & Darrach	#12	Leslie Gulch, Oregon (43°18′26″ N, 117°17′32″ W, 952 m asl)	11 May 2024	124.04	3.643	colorless	2.94
*Lomatium nudicaule* (Nutt.) J.M. Coult. & Rose (voucher WNS-Ln-5374)	#1	Boise Foothills, Idaho (43°32′45″ N, 115°48′15″ W, 1146 m asl)	12 June 2022	191.37	0.564	pale yellow	0.30
*Lomatium nudicaule* (Nutt.) J.M. Coult. & Rose	#2	Boise Foothills, Idaho (43°32′45″ N, 115°48′15″ W, 1146 m asl)	12 June 2022	46.03	0.100	pale yellow	0.22
*Lomatium nudicaule* (Nutt.) J.M. Coult. & Rose	#3	Boise Foothills, Idaho (43°32′45″ N, 115°48′15″ W, 1146 m asl)	12 June 2022	42.06	0.063	pale yellow	0.15
*Lomatium nudicaule* (Nutt.) J.M. Coult. & Rose	#4	Near Prairie, Idaho (43°32′33″ N, 115°48′13″ W, 1142 m asl)	25 May 2023	68.16	1.822	colorless	2.67
*Lomatium nudicaule* (Nutt.) J.M. Coult. & Rose	#5	Near Prairie, Idaho (43°32′33″ N, 115°48′13″ W, 1142 m asl)	25 May 2023	67.14	1.845	colorless	2.75
*Lomatium nudicaule* (Nutt.) J.M. Coult. & Rose	#6	Near Prairie, Idaho (43°32′33″ N, 115°48′13″ W, 1142 m asl)	25 May 2023	49.56	1.253	pale yellow	2.53
*Lomatium nudicaule* (Nutt.) J.M. Coult. & Rose	#7	Near Midvale, Idaho (44°26′45″ N, 116°48′3″ W, 963 m asl)	21 May 2024	120.37	3.620	colorless	3.01
*Lomatium packardiae* Cronquist (voucher WNS-Lpack-0173)	#1	Near Midvale, Idaho (44°25′29″ N, 116°49′19″ W, 988 m asl)	21 May 2024	70.22	1.287	colorless	1.83
*Lomatium packardiae* Cronquist	#2	Near Midvale, Idaho (44°26′42″ N, 116°48′1″ W, 963 m asl)	21 May 2024	123.10	1.920	colorless	1.56
*Lomatium papilioniferum* J.A. Alexander & Whaley (voucher WNS-Lpap-6926)	#1	Between Boggs Junction and Arlington, Oregon (45°41′23″ N, 120°30′0″ W, 97 m asl)	17 April 2023	90.10	0.179	yellow	0.20
*Lomatium papilioniferum* J.A. Alexander & Whaley	#2	Between Boggs Junction and Arlington, Oregon (45°41′23″ N, 120°30′0″ W, 97 m asl)	17 April 2023	134.46	1.532	yellow	1.14
*Lomatium papilioniferum* J.A. Alexander & Whaley	#3	Between Boggs Junction and Arlington, Oregon (45°41′23″ N, 120°30′0″ W, 97 m asl)	17 April 2023	112.70	2.208	colorless	1.96
*Lomatium papilioniferum* J.A. Alexander & Whaley	#4	Between Boggs Junction and Arlington, Oregon (45°41′23″ N, 120°30′0″ W, 97 m asl)	17 April 2023	153.02	2.349	pale yellow	1.54
*Lomatium papilioniferum* J.A. Alexander & Whaley	#5	Near Mann Creek Reservoir, Idaho (44°23′43″ N, 116°53′45″ W, 900 m asl)	21 May 2024	71.85	2.066	yellow	2.88
*Lomatium papilioniferum* J.A. Alexander & Whaley	#6	Near Mann Creek Reservoir, Idaho (44°23′43″ N, 116°53′45″ W, 900 m asl)	21 May 2024	68.18	2.199	yellow	3.23
*Lomatium papilioniferum* J.A. Alexander & Whaley	#7	Near Mann Creek Reservoir, Idaho (44°24′5″ N, 116°53′53″ W, 883 m asl)	21 May 2024	90.42	2.754	yellow	3.05
*Lomatium papilioniferum* J.A. Alexander & Whaley	#8	Near Mann Creek Reservoir, Idaho (44°24′4″ N, 116°53′53″ W, 884 m asl)	21 May 2024	62.27	2.071	yellow	3.33
*Lomatium triternatum* (Pursh) J.M. Coult. & Rose var. *triternatum* (voucher WNS-Ltt-7101)	#1	Near Prairie, Idaho (43°30′25″ N, 115°55′35″ W, 1460 m asl)	25 May 2023	30.30	0.538	colorless	1.77
*Lomatium triternatum* (Pursh) J.M. Coult. & Rose	#2	Near Prairie, Idaho (43°30′25″ N, 115°55′35″ W, 1460 m asl)	25 May 2023	39.22	0.638	colorless	1.63
*Lomatium triternatum* (Pursh) J.M. Coult. & Rose	#3	Near Prairie, Idaho (43°30′25″ N, 115°55′35″ W, 1460 m asl)	25 May 2023	27.06	0.367	colorless	1.36
*Lomatium triternatum* (Pursh) J.M. Coult. & Rose	#4	Near Arrowrock Reservoir, Idaho (43°36′41″ N, 115°49′59″ W, 984 m asl)	9 May 2024	87.12	1.569	pale yellow	1.80
*Lomatium triternatum* (Pursh) J.M. Coult. & Rose	#5	Near Arrowrock Reservoir, Idaho (43°36′42″ N, 115°49′56″ W, 985 m asl)	9 May 2024	48.25	1.038	colorless	2.15

## Data Availability

All data are available in this report. Additional information is available from the corresponding author upon reasonable request.

## Supplementary Materials

The following supporting information can be downloaded at https://www.mdpi.com/article/10.3390/plants14020186/s1: Table S1: Instrument details for the gas chromatographic analyses of *Lomatium* species.
